# Effect of tetrahedral framework nucleic acids on the reconstruction of tendon‐to‐bone injuries after rotator cuff tears

**DOI:** 10.1111/cpr.13605

**Published:** 2024-01-28

**Authors:** Pinxue Li, Liwei Fu, Chao Ning, Jiang Wu, Zizheng Xu, Zhiyao Liao, Cangjian Gao, Xiang Sui, Yunfeng Lin, Shuyun Liu, Zhiguo Yuan, Quanyi Guo

**Affiliations:** ^1^ Institute of Orthopedics Chinese PLA General Hospital, the First Medical Center, Beijing Key Laboratory of Regenerative Medicine in Orthopedics, Key Laboratory of Musculoskeletal Trauma & War Injuries PLA Beijing People's Republic of China; ^2^ School of Medicine Nankai University Tianjin People's Republic of China; ^3^ State Key Laboratory of Oral Diseases, National Clinical Research Center for Oral Diseases, West China Hospital of Stomatology Sichuan University Chengdu People's Republic of China; ^4^ Department of Bone and Joint Surgery, Renji Hospital School of Medicine, Shanghai Jiaotong University Shanghai People's Republic of China

## Abstract

Clinicians and researchers have always faced challenges in performing surgery for rotator cuff tears (RCT) due to the intricate nature of the tendon‐bone gradient and the limited long‐term effectiveness. At the same time, the occurrence of an inflammatory microenvironment further aggravates tissue damage, which has a negative impact on the regeneration process of mesenchymal stem cells (MSCs) and eventually leads to the production of scar tissue. Tetrahedral framework nucleic acids (tFNAs), novel nanomaterials, have shown great potential in biomedicine due to their strong biocompatibility, excellent cellular internalisation ability, and unparalleled programmability. The objective of this research was to examine if tFNAs have a positive effect on regeneration after RCTs. Experiments conducted in a controlled environment demonstrated that tFNAs hindered the assembly of inflammasomes in macrophages, resulting in a decrease in the release of inflammatory factors. Next, tFNAs were shown to exert a protective effect on the osteogenic and chondrogenic differentiation of bone marrow MSCs under inflammatory conditions. The in vitro results also demonstrated the regulatory effect of tFNAs on tendon‐related protein expression levels in tenocytes after inflammatory stimulation. Finally, intra‐articular injection of tFNAs into a rat RCT model showed that tFNAs improved tendon‐to‐bone healing, suggesting that tFNAs may be promising tendon‐to‐bone protective agents for the treatment of RCTs.

## Graphical abstract

Intra‐articular injection of tFNAs into a rat rotator cuff tear (RCT) model showed that tetrahedral framework nucleic acid (tFNAs) improved tendon‐to‐bone healing, suggesting that tFNAs may be promising tendon‐to‐bone protective agents for the treatment of RCTs.

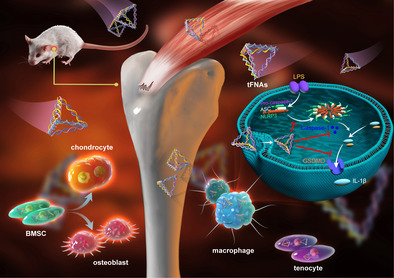

## 1 INTRODUCTION

The poor long‐term results of rotator cuff tear (RCT) surgery, caused by the intricate tendon‐bone gradient structure, often lead to high retearing rates, which will ultimately cause substantial pain and a heavy economic burden for patients.^1^, ^2^ The tendon‐bone junction structure of the normal rotator cuff contains the layer‐by‐layer evolution of tendon, cartilage, and bone, which in turn includes different cellular and matrix components, the arrangement of collagen fibres, and varying mechanical properties.^3^, ^4^ After the tendon‐bone junction sustains damage, the body faces challenges in maintaining a balanced growth of different tissue types within the gradient structure.^5^, ^6^ In addition, the generation of an inflammatory microenvironment further aggravates tissue damage and negatively affects the regeneration process of MSCs, which eventually leads to the production of scar tissue.^7^, ^8^ Promising treatment approaches for tendon‐bone injury, such as tissue‐engineered tendons with gradient structures, mesenchymal stem cell (MSCs) therapy, and allotransplantation,^9^, ^10^, ^11^, ^12^ have been suggested due to advancements in tissue engineering and regenerative medicine. However, considering the intricate biological structure of the tendon bone and the lack of understanding of the regenerative mechanism, the ideal treatment approach to restore the original structure and function after RCTs needs further exploration.^13^, ^14^, ^15^

Recently, tetrahedral framework nucleic acids (tFNAs), a type of nanomaterials composed of four prearranged single‐stranded DNAs, have recently demonstrated significant promise in the biomedical domain due to their favourable biocompatibility, exceptional cellular uptake, and unparalleled programming.^16^, ^17^, ^18^, ^19^, ^20^ Tian et al. proved that tFNAs can have the ability to penetrate cells via caveolin‐mediated endocytosis, subsequently impacting diverse biological processes in receptor cells.^21^ Research has indicated that tFNAs not just stimulate cell growth, movement, and specialisation but also have a significant function in regulating inflammation.^22^, ^23^ In particular, Gao et al. found that tFNAs could enhance the proliferation of myoblasts and skeletal muscle regeneration after acute muscle injury.^24^ Shao et al. confirmed that tFNAs have the ability to stimulate the growth and encourage the development of MSCs towards bone formation.^25^ In addition, our group suggested in previous research that tFNAs have the potential to enhance the chondrogenic differentiation of synovium‐derived MSCs and accelerate articular cartilage regeneration in vivo.^26^ Zhang et al. found that a cascade of polyphenol‐mediated framework nucleic acid‐based responsiveness in psoriasis dissolves nanocomplexes.^27^ Moreover, Jiang et al. carried through successful synthesis of miR mimic, miR‐27 to upgrade the traditional tFNAs design, which have excellent effect for treatment of skin fibrosis.^28^ Furthermore, recent evidence revealed that tFNAs can inhibit the release of cytokines such as IL‐6, TNF‐α, and IL‐1β in periodontal ligament stem cells and thus play a protective role against the inhibition of osteogenic differentiation in the inflammatory microenvironment.^29^ In conclusion, considering the destruction of the tendon‐bone structure in RCTs, tFNAs may be an ideal drug for gradient tissue regeneration in the resulting inflammatory environment. Until now, there have been no investigations examining the impact of tFNAs on the reconstruction of the tendon‐to‐bone in the rotator cuff.

**FIGURE 1:**
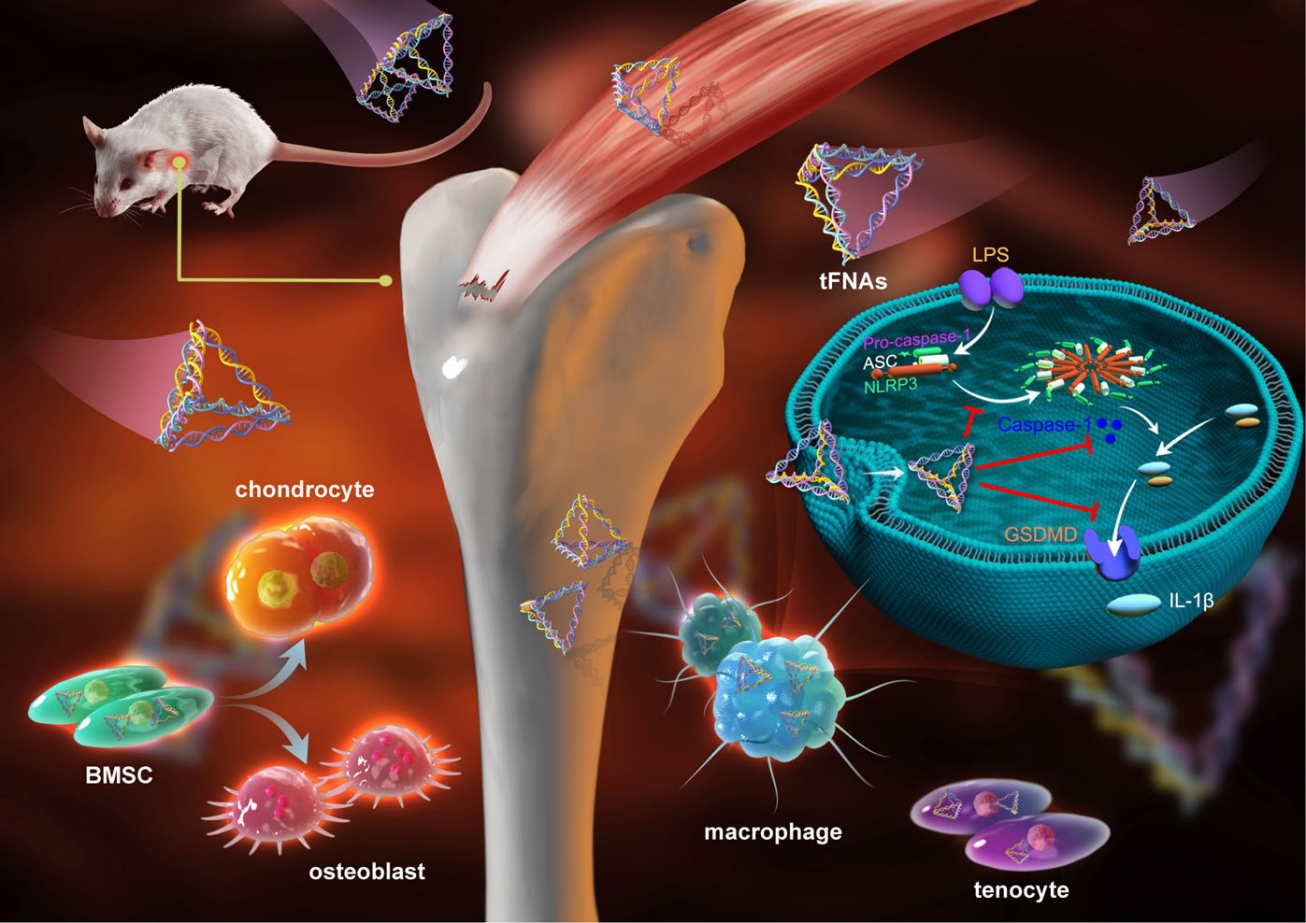
The use of tFNAs improves the healing process of tendon‐to‐bone injuries following rotator cuff tears.

In this study (Figure 1), we first explored the regulatory effect of tFNAs on inflammasomes in macrophages. Next, in vitro, experiments were conducted to determine whether tFNAs could play a protective role in the osteogenic and chondrogenic differentiation of MSCs under inflammatory conditions. Furthermore, we also explored the protective effect of tFNAs on tendon‐associated protein expression levels in tenocytes after inflammatory stimulation. Finally, we administered tFNAs into the joint cavity of a rat RCT model to investigate the ability of tFNAs to enhance tendon‐bone healing. The results showed that tFNAs improve tendon‐bone healing, indicating that they might be a promising tendon‐bone protective agent for RCT treatment.

## 2 MATERIALS AND METHODS

### 2.1 Production and characterisation of tFNAs

Four ssDNAs^30^ (Table 1), which were well‐designed according to the methods described in previous research, were combined in TM buffer, subjected to denaturation at 95°C, and then annealed at 4°C. Initially, we employed dynamic light scattering (DLS) for the determination of the mean size and electrical charge of the particles. The morphology of the tFNAs was confirmed using atomic force microscopy (AFM). To ascertain the molecular weights of the tFNAs, the technique of polyacrylamide gel electrophoresis (PAGE) was employed. To further validate the arrangement of the tFNAs, transmission electron microscopy (TEM) was employed.

**TABLE 1:** The sequences of the ssDNAs.

ssDNA	Base sequence
S1	5′‐ATTTATCACCCGCCATAGTAGACGTATCACCAGGCAGTTGAGACGAACATTCCTAAGTCTGAA‐3′
S2	5′‐ ACATGCGAGGGTCCAATACCGACGATTACAGCTTGCTACACGATTCAGACTTAGGAATGTTCG‐3′
S3	5′‐ACTACTATGGCGGGTGATAAAACGTGTAGCAAGCTGTAATCGACGGGAAGAGCATGCCCATCC‐3′
S4	5′‐ACGGTATTGGACCCTCGCATGACTCAACTGCCTGGTGATACGAGGATGGGCATGCTCTTCCCG‐3′
Cy3‐S1	5′Cy3‐ATTTATCACCCGCCATAGTAGACGTATCACCAGGCAGTTGAGACGAACATTCCTAAGTCTGAA‐3′

### 2.2 Cell culture and treatment

In the in vitro experiments, RAW 264.7 cells, bone marrow mesenchymal stem cells (BMSCs), and tendon cells (tenocytes) were employed. The PLA General Hospital's Institutional Animal Care and Use Committee granted approval for the animal experiments. The RAW 264.7 cells were acquired from the American Type Culture Collection (Bethesda, MD, USA). BMSCs were isolated and cultured according to previous studies.^31^ Briefly, the tibia and femur bones of SD rats were separated, and the bone marrow was washed out of the marrow cavity and transferred to culture flasks. In order to showcase a thriving culture, we conducted an experiment inducing trilineage differentiation to confirm the differentiation capacity of BMSCs. Furthermore, the flow cytometry technique was employed to identify the surface markers of BMSCs. Tenocytes were extracted and grown in accordance with prior investigations.^32^, ^33^ To summarise, samples of tendon tissue were acquired from the Achilles tendon of SD rats, fragmented, digested, centrifuged, resuspended, and subsequently transferred to culture flasks for cultivation. Supplementary Material Sections 1.1 and 1.2 contain comprehensive details of the experimental procedures.

To carry out further experiments, the cells were categorised into three sets: (i) the control group without any treatment; (ii) the LPS group treated with lipopolysaccharide (LPS) at a concentration of 2 μg/mL from Sigma‐Aldrich, USA; and (iii) the tFNAs groups, which were exposed to tFNAs at a concentration of 250 nM along with LPS (2 μg/mL) after being pre‐treated with tFNAs (250 nM) for 1 h.

### 2.3 The internalisation of tFNAs by cells

To confirm the internalisation of tFNAs by RAW 264.7 cells, BMSCs, and tenocytes, we treated tFNAs and ssDNA with cyanine3 (Cy3). Following the separate coculturing of the tFNAs with the three distinct cell types, the fluorescence microscope (Nikon, Japan) was used to capture images. Supplementary Material Section 1.3 contains comprehensive details of the experimental procedures.

### 2.4 Effect of tFNAs on RAW 264.7 cells under inflammatory conditions

Under inflammatory conditions, the gene expression of iNOS, CD206, IL‐1β, and IL‐6 in RAW264.7 cells was detected using RT‐qPCR. In order to investigate the impact of tFNAs on the assembly of inflammasomes during inflammatory circumstances, we utilised Western blotting to identify the protein levels of ACS, NLRP3, pro‐caspase‐1, caspase‐1, and IL‐1β in RAW264.7 cells. The Supplementary Material Section 1.4 provides a thorough explanation of the experimental procedures.

### 2.5 The impact of tFNAs on the osteogenic differentiation of BMSCs in the presence of inflammation

In order to demonstrate the impact of tFNAs on the osteogenic differentiation of BMSCs during inflammatory circumstances, we used osteogenic induction medium (Cyagen Biosciences, Guangzhou, China). After 14 days of coculture in three different media, Alizarin red staining was used to observe calcium nodule deposition to evaluate the degree of osteogenesis. RT‐qPCR was used to analyse the gene expression of ALP, OPN, and RUX2, which are associated with the osteogenic differentiation of BMSCs, in various groups following a 1‐day culture. After being treated with tFNAs or LPS for a duration of 3 days, Western blotting and immunofluorescence staining were conducted to detect the intracellular distribution and protein expression of OPN and RUNX2 in BMSCs. Supplementary Material Section 1.5 contains comprehensive details of the experimental procedures.

### 2.6 The impact of tFNAs on the chondrogenic differentiation of BMSCs during inflammatory circumstances

In order to investigate the impact of tFNAs on the osteogenic differentiation of BMSCs in the presence of inflammation, we created BMSC pellets in a laboratory setting using the methodology described in our prior research.^34^ After 21 days of coculture in the three distinct media, the level of chondrogenesis was evaluated using haematoxylin‐eosin (H&E), Alcian blue, Safranin O, and immunohistochemical staining for type II collagen (Col II). RT‐qPCR was used to detect the gene expression of SOX‐9, Aggrecan, Col II, and type I collagen (Col I). In conclusion, we additionally investigated the detection of chondrogenesis‐associated proteins through Western blot analysis. Supplementary Material Section 1.6 contains comprehensive details of the experimental procedures.

### 2.7 Effect of tFNAs on tenocyte protein expression under inflammatory conditions

In order to investigate the impact of tFNAs on the expression of tendon‐associated indicators (COL I, COL III, and TNMD) during inflammatory circumstances, gene expression of Col I and Col III was detected using RT‐qPCR. Tenocytes were subjected to Western blotting to assess the protein expression levels of Col I, type III collagen (Col III), and tenomodulin (TNMD). Furthermore, immunofluorescent staining was employed to identify the localization and manifestation of Collagen I and Collagen III in tenocytes during inflammatory circumstances. For a more comprehensive understanding of the experiment, please refer to Section 1.7 of the Supplementary Material for detailed procedural information.

### 2.8 Tendon‐to‐bone reconstruction studies after RCT in vivo

A total of 40 male Sprague–Dawley rats weighing between 220 and 240 g were assigned randomly to three groups: the sham group (consisting of 8 rats), the negative control group (consisting of 16 rats), and the tFNAs group (consisting of 16 rats). The surgical procedures are described briefly as follows: the rats were anaesthetised, and the deltoid muscles were exposed after disinfecting the skin. Then, the deltoid of the right shoulder was cut, and the supraspinatus was exposed. Absorbable sutures were used to mark the supraspinatus tendon, followed by transecting the tendon‐to‐bone junction. To stimulate the bone marrow, the greater tubercle of the humerus was ground using a blade. Additionally, a tunnel was drilled at the greater tuberosity of the humerus using an angled puncture needle. In the end, the supraspinatus tendon and the larger tuberosity were reattached using sutures that can be absorbed by the body. Following the procedure, the tFNAs group was administered an intra‐articular injection of 50 μL tFNAs (250 nM) every alternate day, for a duration of 2 weeks, totaling 7 injections. Conversely, the control group received an equivalent amount of normal saline through injection. Samples were collected following euthanasia at 6 and 12 weeks post‐surgery. Following the sampling process, the healing effect was assessed using small‐animal MRI and microcomputed tomography (micro‐CT) scanning. In the biomechanical test, the sample was fixed in a gripping device and mounted on a mechanical testing machine, and the sample was gradually stretched at a speed of 3 mm/min until fracture. Ultimately, the final load and rigidity were determined based on the force‐displacement graph. Furthermore, the histological analysis of the regenerated tissues was assessed using H&E, safranin O/FAST green, Masson, and Sirius red staining. To examine the collagen content, immunohistochemical staining was performed using antibodies against Col I and Col III. The regenerative impact on the rotator cuff was assessed based on the histological assessment of the tendon‐bone attachment site and the score for tendon‐tendon evaluation. Supplementary Material Sections 1.8–1.12 contain comprehensive information on the experimental procedures.

### 2.9 Statistical analysis

The variations between groups were examined using either one‐way ANOVA or Student's *t*‐test with the aid of SPSS 18.0 statistical software. The data are presented as the average ± standard deviation (SD), and *p < 0.05 indicates the level of statistical significance.

## 3 RESULTS

### 3.1 Synthesis and characterisation of tFNAs

**FIGURE 2:**
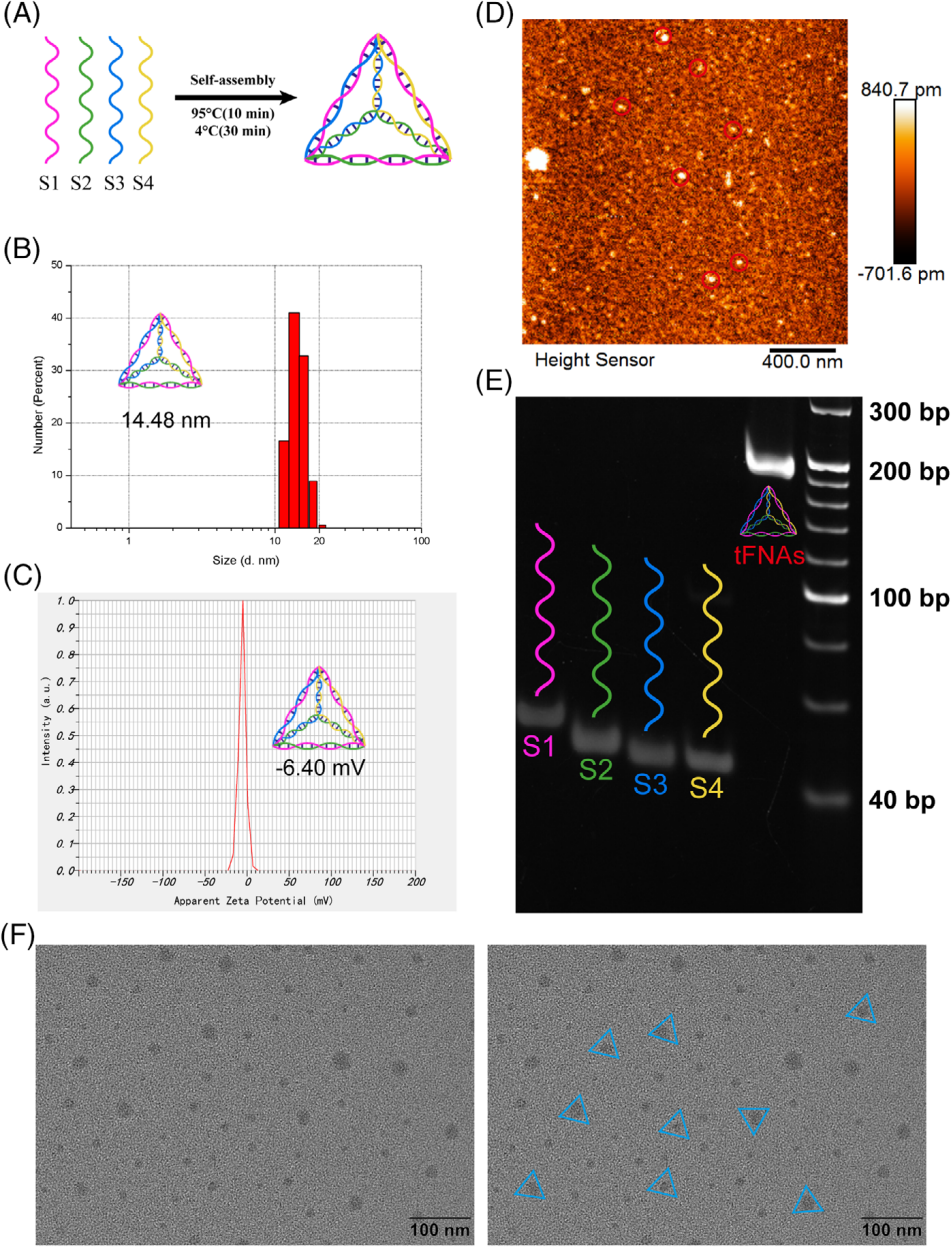
Production and characterisation of tFNAs. (A) S The process of creating tFNAs is illustrated in a schematic diagram. (B) Size distribution of tFNAs. (C) Zeta potential distribution. (D) AFM image. (E) PAGE indicating the molecular weight. (F) The surface morphology and average size observed by TEM imaging.

According to our team's previous report,^26^ we created tFNAs that included four prearranged ssDNAs (Table 1) using the method we had prepared. The triangular structure in Figure 2A was formed by the self‐assembly of each ssDNA, which then combined with the other three single‐stranded structures using the principle of base complementary pairing, resulting in the formation of a stable tetrahedral structure. The mean particle size (Figure 2B) and zeta potential (Figure 2C) of the tFNAs were measured by DLS and were 14.48 ± 3.64 nm and − 6.40 ± 3.24 mV, respectively. The successful synthesis of tFNAs was confirmed using AFM, and the morphology was preliminarily observed (Figure 2D). Finally, we used PAGE and TEM images (Figure 2E, F) to further detect and observe the molecular weight and morphology of the tFNAs. The above results indicated that tFNAs with a uniform size were successfully prepared and were stable in TM buffer.

### 3.2 Cell culture and cellular uptake of tFNAs

**FIGURE 3:**
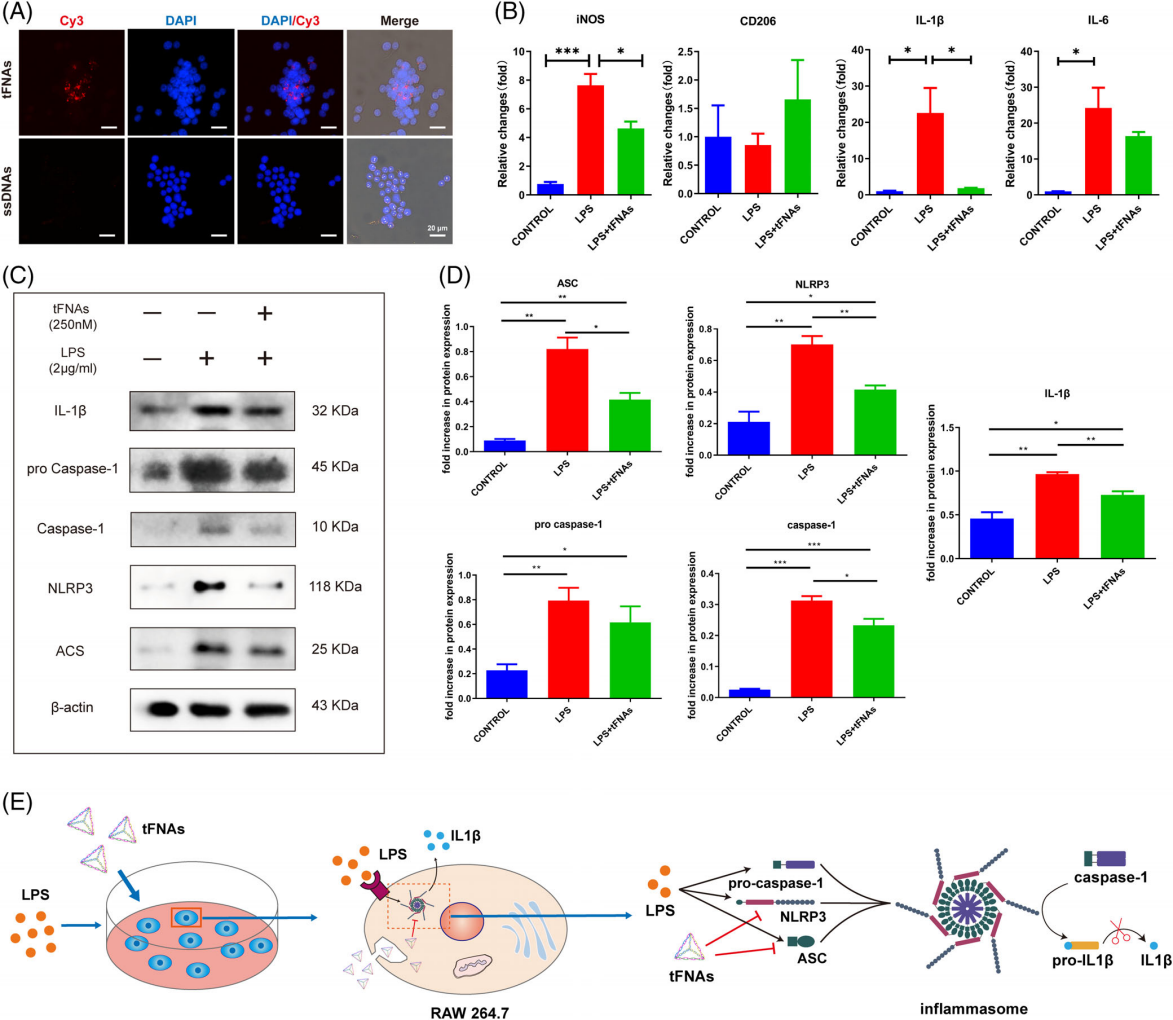
Effect of tFNAs on inflammasome assembly in RAW 264.7 cells under inflammatory conditions. (A) Cellular uptake of ssDNAs and tFNAs. (B) Gene expression of iNOS, CD206, IL‐1β, and IL‐6 in RAW264.7 cells treated with LPS and tFNAs. Data are presented as the mean ± SD (*n* = 3). (C) Protein expression of ACS, NLRP3, pro‐caspase‐1, caspase‐1, and IL‐1β in RAW 264.7 cells treated with LPS and tFNAs. (D) Quantitative analysis of the protein expression levels of ACS, NLRP3, pro‐caspase‐1, caspase‐1 and IL‐1β. Data are presented as the mean ± SD (*n* = 3). (E) Schematic diagram of the inhibition of inflammasome activation by tFNAs. Statistical analysis: *p < 0.05, **p < 0.01.

BMSCs were cultured and demonstrated to possess multidirectional differentiation capacity and express MSC‐specific surface markers (Figures S1, S2). Earlier research has indicated that tFNAs have the ability to penetrate cells through caveolin‐mediated endocytosis. Subsequently, they can impact various signalling pathways, thereby modulating the biological activities of specific cells like MSCs.^35^, ^36^ To determine the cellular internalisation of tFNAs, we conducted immunofluorescence staining using Cy3‐modified tFNAs and ssDNA. When Cy3‐modified tFNAs were cultured with RAW 264.7 cells, BMSCs, and tenocytes, it was observed that RAW 264.7 cells (Figure 3A), BMSCs (Figure S3), and tenocytes (Figure S3) readily internalised tFNAs to a significant degree, whereas minimal uptake of ssDNAs occurred in these cells. tFNAs, which are nucleic acid materials with negative charge, can be effectively internalised by RAW 264.7 cells, BMSCs, and tenocytes without the need for extra carriers. This formed the foundation for our subsequent experiments.

### 3.3 tFNAs can reduce LPS‐induced inflammatory responses and inhibit NLRP3 inflammasome activation in RAW 264.7 cells

According to prior research, tFNAs have been found to hinder the M1 phenotypic polarisation in macrophages while encouraging the M2 phenotypic polarisation.^37^ Under inflammatory conditions, the gene expression of iNOS, CD206, IL‐1β, and IL‐6 in RAW264.7 cells was detected using RT‐qPCR. In LPS‐induced macrophages, the upregulation of the M1 phenotype‐associated gene iNOS and the inflammatory cytokines IL‐1β and IL‐6 was evident, as depicted in Figure 3B. The gene expression of iNOS and IL‐1β in the macrophages pretreated with tFNAs was considerably reduced compared to the LPS group. This implies that prior treatment with tFNAs may decrease the inclination of macrophages to polarise towards the M1 phenotype, consequently diminishing the expression of associated inflammatory markers. The NLRP3 inflammasome consists of NLRP3, ASC, and pro‐caspase1. It has the ability to cleave caspase‐1, leading to the initiation of GSDMD‐mediated pore formation in the cellular membrane and the subsequent liberation of IL‐1β and IL‐18. In order to investigate the impact of tFNAs on the assembly of NLRP3 inflammasome, protein expression of ACS, NLRP3, pro‐caspase‐1, caspase‐1, and IL‐1β in RAW264.7 cells was detected using Western blotting. The LPS group exhibited a substantial increase in the expression of NLRP3 inflammasome‐associated proteins, as depicted in Figure 3C, D. Under conditions of LPS‐induced inflammation, it was observed that the formation of the NLRP3 inflammasome and the generation of IL‐1β were enhanced. The protein expression levels of NLRP3 and ASC were significantly reduced in the tFNAs+LPS group compared to those in the LPS group. Furthermore, the tFNAs+LPS group exhibited notably reduced protein expression levels of caspase‐1 and IL‐1β compared to the LPS group. The findings indicated that tFNAs hindered the formation of the NLRP3 inflammasome by suppressing the production of NLRP3 and ASC, consequently decreasing the secretion of IL‐1β during inflammatory circumstances (Figure 3E). This laid the foundation for our subsequent in vivo tests.

### 3.4 Under inflammatory circumstances, tFNAs facilitated the osteogenic differentiation of BMSCs

**FIGURE 4:**
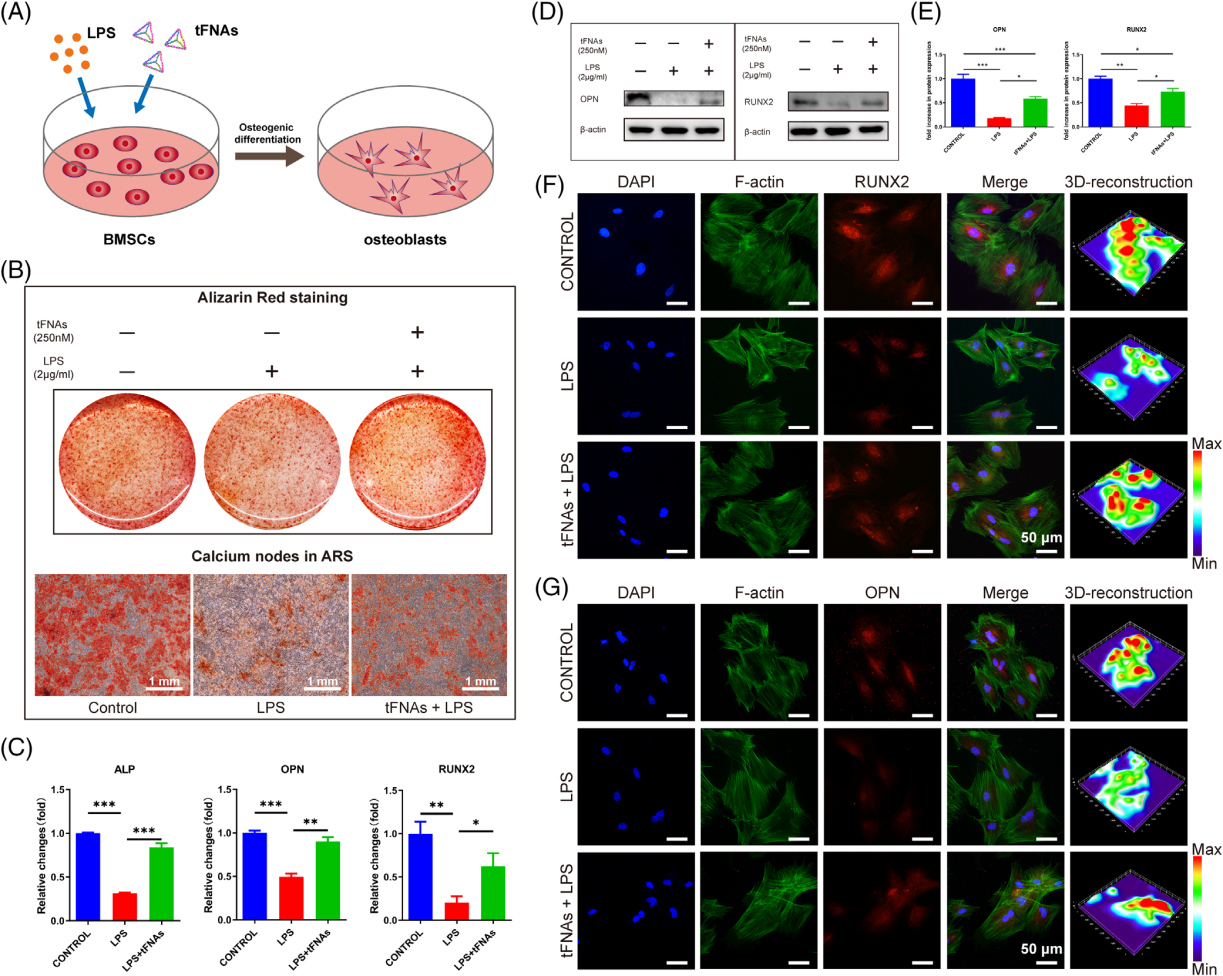
Under inflammatory circumstances, tFNAs enhance the osteogenic differentiation of BMSCs. (A) Schematic diagram of osteogenic differentiation induction in BMSCs treated with LPS and tFNAs. (B) Alizarin red staining was used to detect the osteogenic differentiation of BMSCs following a 14‐day treatment with LPS and tFNAs. (C) Gene expression of ALP, OPN, and RUNX2 in BMSCs treated with LPS and tFNAs for 1 day. (D) WB analysis was performed to examine the levels of RUNX2 and OPN expression in BMSCs after treatment with LPS and tFNAs for a duration of 3 days. (E) Analysing the levels of protein expression for RUNX2 and OPN in BMSCs that were exposed to LPS and tFNAs using quantitative methods. The data are displayed as the average plus standard deviation (*n* = 3). (F), (G). Immunofluorescence detection of RUNX2 and OPN. (Green F‐actin, blue nucleus, and red protein.) The measurement bars have a length of 50 μm. Statistical analysis: *p < 0.05, **p < 0.01, ***p < 0.001.

According to previous research, tFNAs have been found to enhance the osteogenic differentiation of MSCs.^38^, ^39^ According to reports, the capacity of MSCs to undergo osteogenic differentiation is greatly reduced when exposed to inflammatory stimulation induced by LPS.^40^ In order to investigate if tFNAs can protect the osteogenic differentiation of BMSCs during inflammatory conditions (Figure 4A), Alizarin red staining, RT‐qPCR, Western blotting, and immunofluorescence detection were used. After a 14‐day treatment with LPS and tFNAs, the presence of calcium nodules, which are late indicators of osteogenic differentiation, was detected using Alizarin red staining. According to Figure 4B, the LPS group exhibited a notable decrease in the quantity of calcium nodules compared to the control group, while the LPS + tFNAs group demonstrated a higher number of calcium nodules than the LPS group. This demonstrated that the osteogenic differentiation ability of BMSCs was inhibited, and tFNAs treatment could enhance this ability in an inflammatory environment. Afterwards, we examined the impact of tFNAs on the genetic‐level osteogenic differentiation of BMSCs in the presence of inflammation. The results of the RT‐qPCR analysis (Figure 4C) indicated that the inflammatory conditions induced by LPS suppressed the expression of osteogenesis‐related markers (ALP, OPN, and RUNX2) in BMSCs. However, tFNAs mitigated the inhibitory impact of LPS on the gene expression associated with BMSCs. Figure 4D, E shows that the protein contents (OPN and RUNX2) of the LPS group were decreased. Furthermore, the protein concentration in the LPS + tFNAs group exceeded that of the LPS group. The immunofluorescence staining results (Figure 4F, G) indicated a significant decrease in the expression of RUNX2 and OPN in the LPS group compared to the control group. However, the fluorescence intensity of associated proteins in BMSCs was significantly increased in the LPS + tFNAs group compared to the LPS group. The findings indicated that tFNAs provided protection for BMSCs and enhanced their ability to differentiate into osteogenic cells in the presence of inflammation.

### 3.5 tFNAs enhanced the chondrogenic differentiation of BMSCs under inflammatory conditions

**FIGURE 5:**
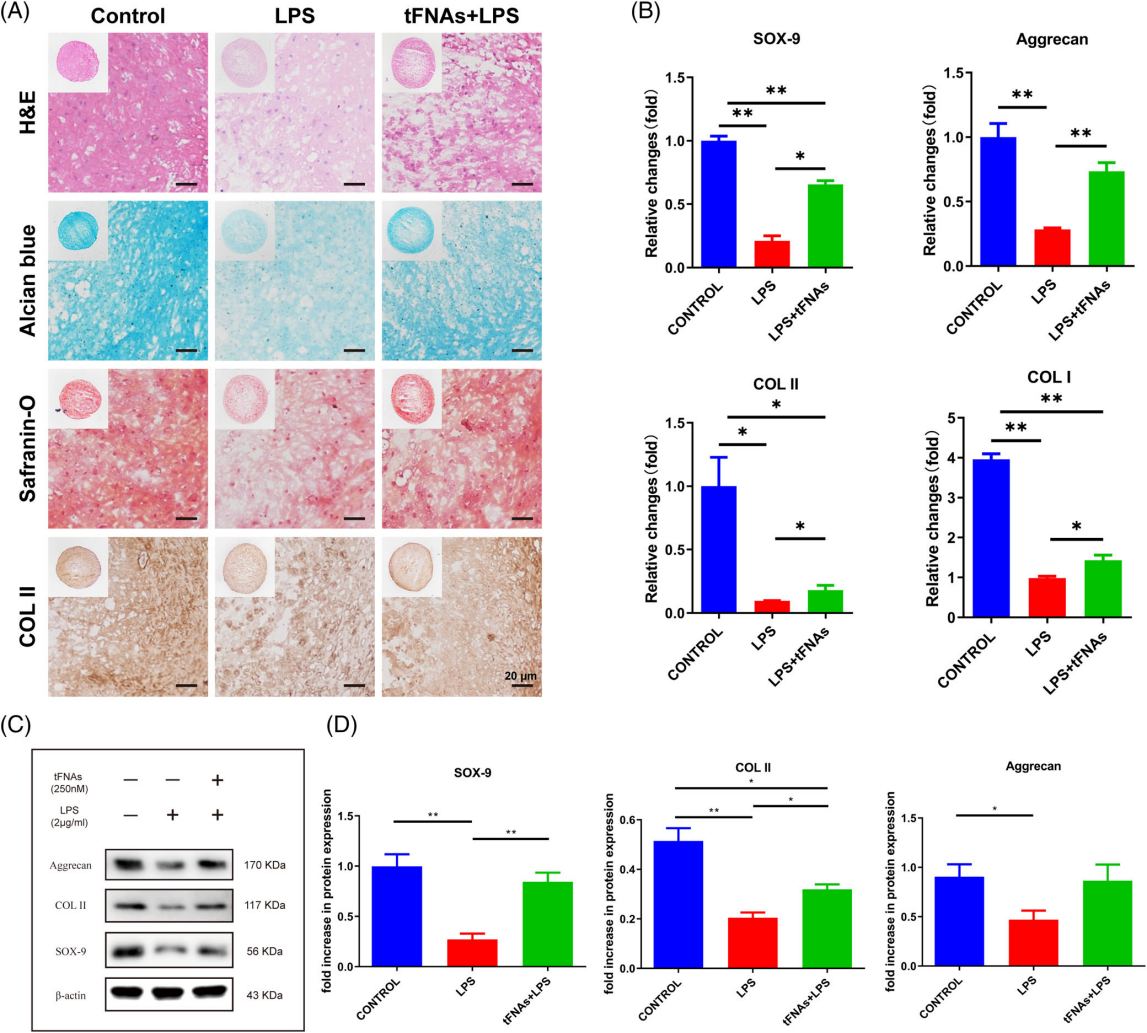
tFNAs enhance the chondrogenic differentiation of BMSCs under inflammatory conditions. (A) H&E, Alcian blue and safranin‐O staining and immunohistochemical analysis of Col II in pellets treated with LPS and tFNAs for 21 days. (B) Gene expression of SOX‐9, Aggrecan, Col II, and Col I (*n* = 3). (C) Analysis of the expression levels of SOX‐9, Aggrecan, and Col II in pellets was conducted by WB. (D) quantitative assessment was conducted on the protein expression levels (*n* = 3). Statistical analysis: *p < 0.05, **p < 0.01.

According to reports, tFNAs have the ability to enhance the chondrogenic differentiation of MSCs when exposed to cartilage induction medium.^26^ Moreover, the chondrogenic differentiation ability of BMSCs is inhibited under inflammatory conditions.^41^ Therefore, the impact of tFNAs on the differentiation of BMSCs into chondrocytes was examined by treating BMSC pellets with LPS and tFNAs in a chondrogenic medium. Initially, the level of chondrogenesis was evaluated through histological examination and immunohistochemical staining of cartilage pellets using Col II. Based on the findings in Figure 5A, our successful preparation of cartilage pellets is confirmed by the H&E staining results, which demonstrate a favourable morphology and even distribution of extracellular matrix, Furthermore, the LPS group exhibited a notable decrease in both cell count and density compared to the control group. The tFNAs + LPS group outperformed the LPS group but was not as good as the control group. The cartilage pellet in each group was assessed for its polysaccharide content using Alcian blue staining and safranin‐O staining. The amount of polysaccharides in the control group's pellets was substantial and consistent, whereas the pellets in the LPS group exhibited a generally faint staining, and the staining of pellets in the tFNAs + LPS group appeared dark externally and pale internally. The Col II immunohistochemical staining yielded comparable results to the staining obtained with Alcian blue and safranin‐O. The control group had the strongest positive staining, the LPS group had the weakest positive staining, and the tFNAs + LPS group had moderate staining. Moreover, the expression of the cartilage‐related genes SOX‐9, Aggrecan, Col II, and Col I was detected by RT‐qPCR, as illustrated in Figure 5B. In the chondrogenic induction environment, the gene expression of SOX‐9, Aggrecan, Col II, and Col I in BMSC pellets was found to be suppressed due to LPS‐induced inflammatory conditions. However, tFNAs treatment resulted in the enhancement of gene expression for these specific genes.

To further verify the protective effect of tFNAs on BMSCs in an inflammatory environment, the expression levels of chondrogenesis‐related proteins were further examined by Western blotting. According to the protein bands and quantitative analysis (Figure 5C, D), it was observed that the LPS group exhibited a notable decrease in the expression of chondrogenesis‐related proteins compared to the control group (*p* < 0.05) during inflammatory conditions. Conversely, the tFNAs + LPS group displayed higher protein expression than the LPS group (*p* < 0.05). Collectively, our findings indicated that tFNAs have the ability to enhance the chondrogenic differentiation of BMSCs in the presence of inflammation.

### 3.6 tFNAs regulated tenocyte protein expression under inflammatory conditions

**FIGURE 6:**
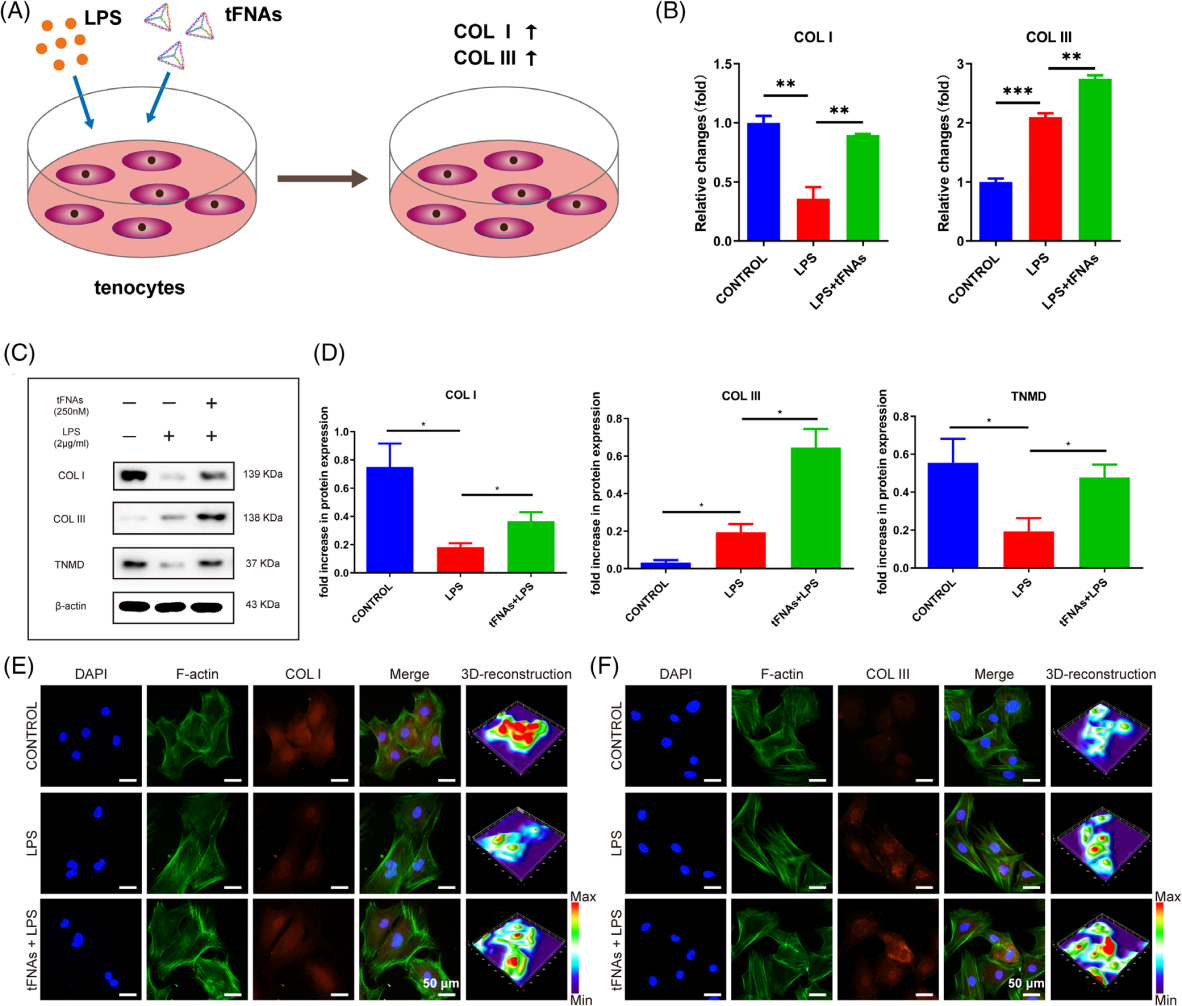
tFNAs regulate tenocyte protein expression under inflammatory conditions. (A) Schematic illustration of the regulation of collagen expression by tFNAs in tenocytes treated with LPS and tFNAs. (B) Gene expression of Col I and Col III in tenocytes treated with LPS and tFNAs for 1 day. (C) WB analysis of the expression levels of Col I, Col III, and TNMD in tenocytes treated with LPS and tFNAs for 3 days. (D) Quantitative analysis of the protein expression levels of Col I, Col III, and TNMD in tenocytes treated with LPS and tFNAs (*n* = 3). (E), (F) Detection of Col I and Col III using immunofluorescence (Green F‐actin, blue nucleus, and red protein). Statistical analysis: *p < 0.05, **p < 0.01, ***p < 0.001.

The impact of tFNAs on tenocytes was examined in the presence of inflammation, as depicted in Figure 6A. RT‐qPCR was used to evaluate the gene expression of Col I and Col III in tenocytes exposed to inflammatory conditions, as shown in Figure 6B. In tenocytes, during inflammatory conditions, the gene expression of Col I showed a significant decrease (*p* < 0.01), while the gene expression of Col III exhibited a significant increase (*p* < 0.001). However, the tFNAs + LPS group showed increased expression of both collagens under inflammatory conditions. Using Western blotting, we assessed the levels of Col I, Col III, and TNMD proteins associated with tendons in tenocytes. As depicted in Figure 6C, D, in normal tenocytes, both Col I and TNMD were highly expressed, while Col III was hardly expressed. During inflammatory conditions, the tenocytes exhibited a significant decrease in the expression of Col I and TNMD (*p* < 0.01), whereas the expression of Col III was increased (*p* < 0.01). The expression of these three proteins in the tFNAs + LPS group was significantly increased (*p* < 0.01) when compared to the LPS group. Moreover, immunofluorescence staining (Figure 6E, F) for Col I and Col III exhibited a similar pattern, with a decrease in fluorescence intensity observed for Collagen I and an increase for Col III in the LPS group. The tFNAs + LPS group showed increased fluorescence intensity of Col I and Col III compared to that in the LPS group. Col I plays the most important role in maintaining normal tendon function, while Col III is mainly present in normal tendon sheaths and diseased tendons. These results suggested that tFNAs could enhance collagen production in tenocytes and increase the expression of the tendon‐related protein TNMD under inflammatory conditions, which opened up possibilities for utilising tFNAs in the regeneration of tendon‐bone junction injuries.

### 3.7 tFNAs promoted tendon‐to‐bone rotator cuff regeneration in vivo

**FIGURE 7:**
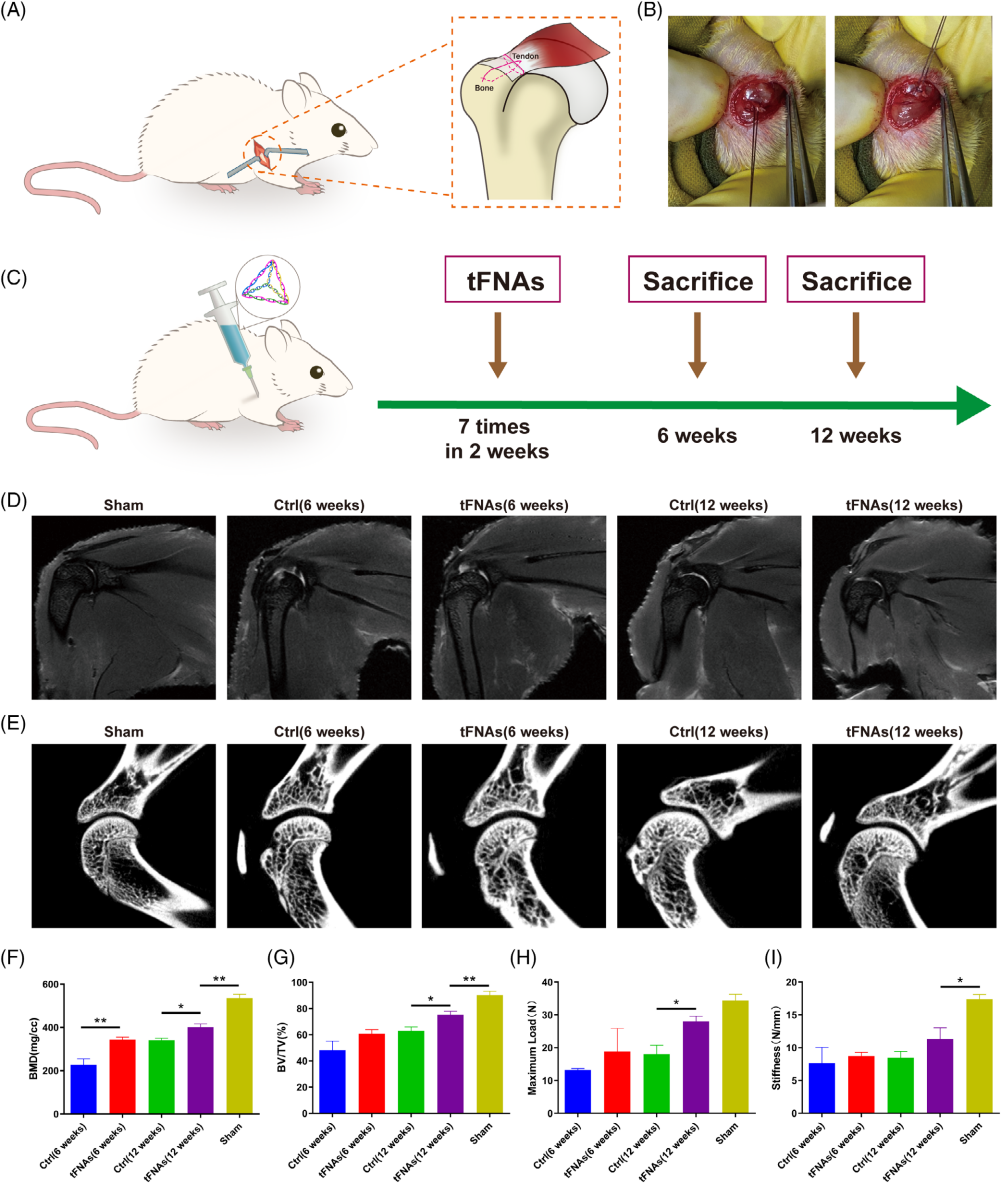
In vivo experimental design and imaging and biomechanical evaluation. (A), (B) Schematic diagram of the rat RCT model. (C) Schematic diagram of the time of intra‐articular injection of tFNAs and the precise time point of sacrifice of experimental rats after the operation. (D) MRI images showing a new tendon in the repaired tissue. (E) Micro‐CT images revealing bone regeneration. (F), (G) Comparative assessment of BMD and BV/TV in different groups (*n* = 4). (H), (I). The maximum load and stiffness values based on the force–displacement curve (*n* = 3). Statistical analysis: *p < 0.05, **p < 0.01.

In order to examine if tFNAs could enhance the healing process of tendon‐bone junction injury, we created an acute rotator cuff injury model in rats, as depicted in Figure 7A, B. A total of 40 male Sprague‐Dawley rats weighing between 220 and 240 g were assigned randomly to three groups: the sham group (*n* = 8), the negative control group (*n* = 16) and the tFNAs group (*n* = 16). After the operation, the tFNAs group received an intra‐articular injection of 50 μL tFNAs (250 nM) every alternate day, amounting to a total of 7 injections spanning 2 weeks. Samples were gathered at 6‐ and 12‐weeks post‐operation (Figure 7C). MRI analysis (Figure 7D) showed that the control group and the tFNAs group at both time points exhibited restoration of the continuity and attachment of the supraspinatus tendon to the tuberosity of the humerus. However, the tFNAs group showed attachment closer to the normal anatomical position than the control group. The tendon fibres of the control group were much more disorganised and thicker than the more arranged fibres of the tFNAs group, indicating rupture of the inner tendon and incomplete repair. In relation to the junction between the tendon and bone, the control group exhibited greater irregularity, while the tFNAs group displayed narrower and tinier regions. In the control group at 6 weeks, bone marrow edema of the humerus head could be observed, but this effect disappeared at 12 weeks. There was no evidence of bone edema in the tFNAs group. Effusion of the shoulder joint could be seen in both groups compared with the control group at two‐time points, indicating the existence of a synovial inflammatory response during the repair process. The synovial inflammatory response in the tFNAs group was less severe, and both groups improved as time lapsed.

The bone regeneration of the humerus's greater tuberosity was assessed using Micro‐CT. According to Figure 7E, the tFNAs group exhibited superior bone regeneration compared to the control group, both at 6‐ and 12‐weeks post‐surgery. Moreover, the BMD and BV/TV values of the regenerated regions were also analysed (Figure 7F, G). At 6‐ and 12‐weeks post‐surgery, the tFNAs group exhibited significantly elevated BMD values compared to the control group. After 12 weeks, the tFNAs group exhibited significantly greater BV/TV values compared to the control group. Nevertheless, at the 12‐week mark following the surgery, there remained a notable distinction (*p* < 0.01) in both BMD and BV/TV measurements between the tFNAs group and the sham‐operated group.

Biomechanical experiments were conducted to validate the mechanical characteristics of the regenerated rotator cuff tissue, with the findings presented in Figure 7H, I. The final capacity and rigidity were determined based on the force–displacement graph. At 12 weeks, the findings indicated that the tFNAs group exhibited a significantly (*p* < 0.01) greater maximum load compared to the control group. At the 12th week, there was no significant difference in the maximum load between the tFNAs group and the sham group. The stiffness values did not show any notable disparity between the tFNAs group and the control group at the two time points. However, both groups exhibited a significant distinction (*p* < 0.01) from the sham group. This indicated that the regenerated tissue was structurally different from the normal tissue and had a lower resistance to deformation.

**FIGURE 8:**
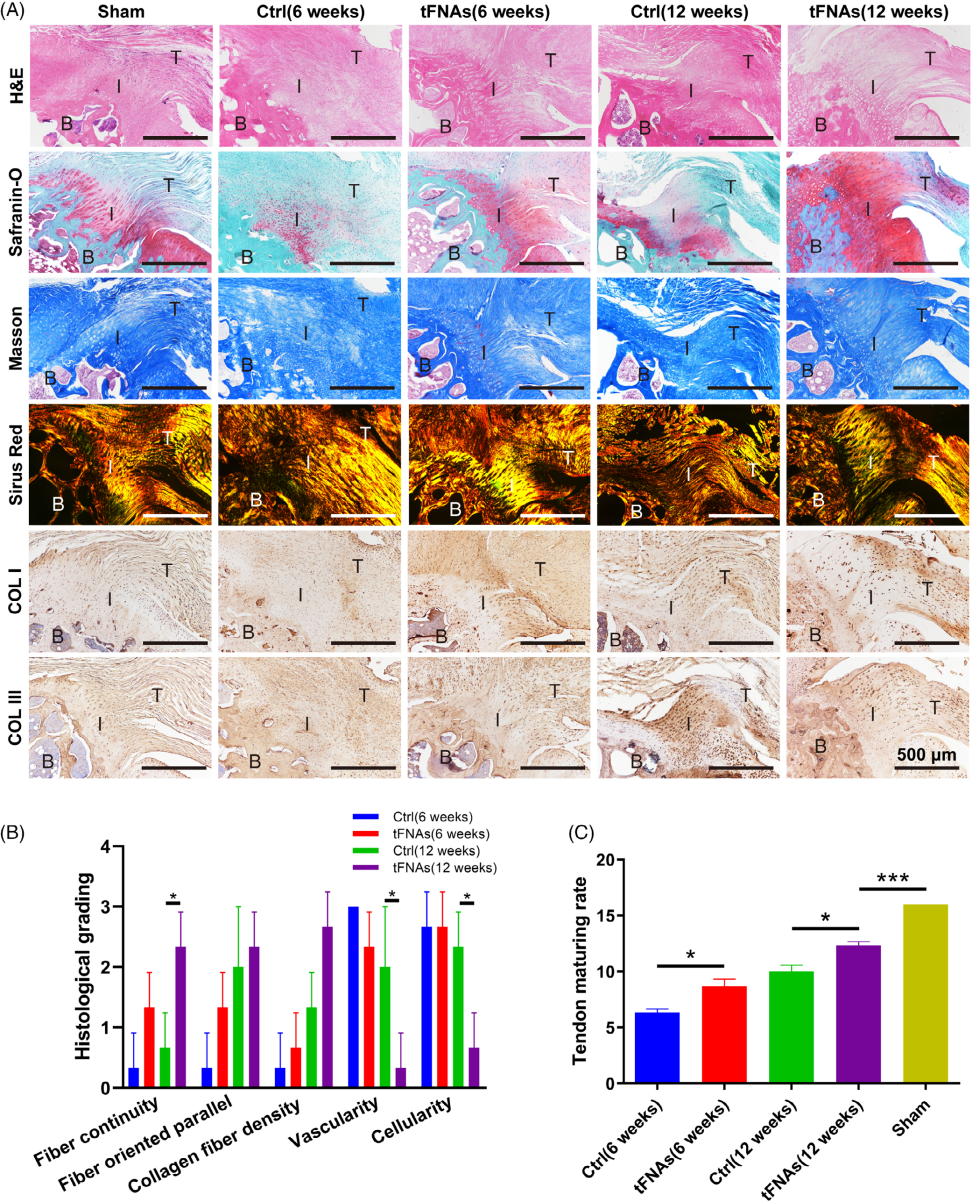
Histological analysis in vivo. (A), (B) At 6 weeks and 12 weeks, the regenerated area was examined using histological techniques including H&E, safranin‐O, Masson, and Sirius red staining, as well as immunohistochemical analyses of collagen I and collagen III. (B: bone; I: interface; T: tendon). (B) Histological scores at the tendon‐bone insertion site after 6 and 12 weeks. (C) Tendon maturation scores in the different groups at 6 and 12 weeks (*n* = 3). Statistical analysis: **p < 0.01.

On the other hand, to evaluate the tissue architecture of the tendon‐bone junction of the regenerated rotator cuff after tFNAs treatment, the histology of the regenerated tissues was evaluated by H&E, safranin O/FAST green, and Masson Sirius red staining and immunohistochemical staining of Col I and Col III, as shown in Figure 8A. In the sham group, neatly arranged collagen fibres were present in the fibrocartilage region and subsequently in the bone region. At 6 weeks, HE staining showed that the repaired tendon in the simple suture group had not healed. A significant quantity of inflammatory cells had infiltrated the area. Fibrocartilaginous cells were almost absent, blood vessels had proliferated, collagen fibres were discontinuous, and their shape was consistent with a scar tissue state: loosely arranged and irregular. In the tFNAs group, there was less inflammatory cell infiltration in the repair area, with fibrochondroid cells and organised collagen fibres. Immature granulation tissue was found at the tendon‐bone interface in both groups. In the 12th week, the repair process was complete, and almost no inflammatory cells were seen in any group. In the control group, some fibrochondroid cells and regenerated fibrocartilage tissue were found, and the collagen fibres were arranged in a relatively orderly manner. However, the tFNAs group showed wide fibrocartilage tissue, the tendon‐bone interface was obvious, and the anatomical structure was closer to that of the sham group. Safranin‐O staining was used to evaluate polysaccharide deposition at the tendon‐bone interface. At 6 weeks, the control group had only a small amount of scattered polysaccharide, while the tFNAs group showed good polysaccharide deposition at 6 weeks. At 12 weeks, the control group still showed uneven staining, while the tFNAs group had a large amount of polysaccharide. The morphology of collagen fibres at the repaired tendon‐bone interface was evaluated by Masson and Sirius red staining. The collagen fibres and fibrocartilage in each group matured further with time. At 6 weeks after the operation, collagen deposition at the tendon‐bone interface was enhanced, the morphology of fibrocartilage was uneven, and the overall morphology was immature and irregular. Twelve weeks after the operation, the collagen fibres were relatively parallel in orientation, widely distributed in the tissue structure, mature and complete, and the morphology and tissue structure of fibrochondrocytes were normal. Compared with the control group, the tFNAs group showed a better repair effect at 6 and 12 weeks, but the effect was worse than that of the sham operation group. To assess the expression and distribution differences of Col I and Col III between groups, we performed immunohistochemical staining, as shown in Figure 8A. In general, Col I was mainly found in normal tendons, while Col III was mainly found in diseased tendon and tendon sheaths. Compared with those in the control group, the content and distribution of Col I in the repaired tendon‐bone in the tFNAs group were higher and denser, and the distribution of collagen fibres was more orderly, which was more similar to the native tissue. However, the control group expressed abundant Col III at 12 weeks. These results indicated that the tFNAs treatment promoted the regeneration and arrangement of collagen at the tendon‐bone interface and the remodelling of the new cartilage‐like tissue.

At 12 weeks, the histologic grading of the tendon‐bone insertion site (Figure 8B) indicated that the tFNAs group exhibited greater collagen fibre continuity and reduced vascularization and cellular presence compared to the control group. Based on the tendon maturation score (Figure 8C), the tFNAs group exhibited significantly greater tendon maturity compared to the control group after 6 and 12 weeks. However, the tendon maturity in the tFNAs group was still considerably lower than that in the sham group. In conclusion, the results of the in vivo experiments suggested that tFNAs are a very promising strategy for the treatment of acute RCT.

## 4 DISCUSSION

In this study, tFNAs, novel nanomaterials, were fabricated as protective agents for healing of the tendon‐bone interface after RCTs. The results showed that tFNAs are a new therapeutic option for tendon‐bone junction healing in an acute RCT model. In vitro experiments showed that tFNAs could simultaneously protect the osteogenic and chondrogenic differentiation of MSCs and the phenotype of tenocytic cells under inflammatory stimulation. In addition, we also demonstrated that tFNAs could simultaneously induce tendon, bone, and cartilage regeneration in vivo, effectively promoting tendon‐bone healing.

As a typical gradient microstructure, the tendon‐bone interface consists of a gradient structure of tendon, cartilage, and bone. RCTs after surgery often occur because gradient tissue regeneration is not synchronised under the interference of inflammatory factors, eventually leading to the generation of scar tissue.^7^, ^8^ Therefore, the simultaneous regeneration of the tendon‐bone gradient structure and regulation of inflammation are very important for ideal regeneration after RCTs.^42^ At present, researchers in the field of tissue engineering and regenerative medicine use biological mesh or gradient scaffold methods most often.^43^, ^44^ Although some food effects have been achieved, these methods often fail to take into account the synchronous regeneration of gradient microstructures. In addition, the aggravation caused by inflammatory factors is not well regulated.^45^ Therefore, a new therapeutic approach is needed to enhance the synchronous regeneration of the tenoskeletal gradient microstructure in inflammatory environments.

We focused on a new type of nanomaterial, tFNAs, that have recently shown great potential in the field of regenerative medicine.^26^ According to previous studies, tFNAs can promote the regeneration of various tissues, including nerves,^46^ bone,^38^ muscle^24^ and cartilage,^26^ by regulating the function of receptor cells and demonstrate a strong protective ability for tissue regeneration in an inflammatory environment. For example, Zhou et al. revealed that tFNAs can inhibit the release of cytokines such as IL‐6, TNF‐α, and IL‐1β in periodontal ligament stem cells and thus exhibit a protective role on osteogenic differentiation in an inflammatory microenvironment.^29^ However, no experiment has investigated whether tFNAs have a positive effect on tendon‐bone tissue healing after RCTs. We hypothesised that such nanomaterials that promote multi‐tissue regeneration may provide a new strategy for the treatment of RCTs.

Therefore, we prepared tFNAs according to the method previously reported by our research group and verified their size and morphology (Figure 2). Then, regulation of the inflammasome in macrophages was used to evaluate the ability of tFNAs to regulate inflammation. The NLRP3 inflammasome is a protein complex composed of NLRP3, ASC, and pro‐caspase1, which can cleave caspase‐1 to trigger GSDMD‐mediated pore formation in the cell membrane and the release of IL‐1β and IL‐18, ultimately leading to cell pyroptosis, which plays a vital role in the regulation of inflammation during tissue regeneration.^47^ In our experiment, when macrophages were cultured in medium containing tFNAs, tFNAs could not only be engulfed by macrophages in large numbers but also reduce the generation of the NLRP3 inflammasome through the classical caspase‐1 pathway under the stimulation of LPS inflammation. Finally, the secretion of inflammatory factors such as IL‐1β was effectively inhibited (Figure 3).

Previous studies have demonstrated that tFNAs can promote the osteogenic and chondrogenic differentiation of MSCs.^25^, ^26^ We then explored the effect of tFNAs on the osteogenic and chondrogenic differentiation of MSCs under LPS inflammatory stimulation in vitro. According to the experimental results, under LPS stimulation, the osteogenic and chondrogenic differentiation abilities of MSCs were inhibited, which indicated that the regeneration process of bone and cartilage tissue at the tendon‐bone interface may be disturbed in the inflammatory microenvironment. After the addition of tFNAs, the osteogenic and chondrogenic effects were significantly enhanced, and the expression of related proteins was also enhanced. Therefore, tFNAs exert clear protective effects on bone and cartilage regeneration by MSCs in an inflammatory environment (Figures 4 and 5). Furthermore, we also injected tenocytes into an inflammation model and found that the expression of tenocyte‐related protein markers was regulated, and tFNAs also showed a significant protective effect (Figure 6). In conclusion, for the three most important tissues of the tendon‐bone junction structure, tFNAs can promote tissue repair and regeneration in an inflammatory environment, making them good candidates for the ideal RCT treatment we discussed above.

To investigate the effect of tFNAs on tendon‐bone regeneration in vivo, we established an acute RCT model using rats. After surgery, 250 nM tFNAs were injected into the shoulder cavity of the rats, and the same volume of normal saline was injected into the control group. According to the MRI and micro‐CT imaging results, both groups at the two‐time points showed restored continuity and attachment of the supraspinatus tendon to the tuberosity of the humerus. However, compared with that in the control group, the inflammatory response in the experimental group was weaker at both time points, and the degree of tissue healing was also better. In addition, we found that the biomechanical parameters of the tFNAs group at 12 weeks showed obvious advantages, with values close to those of normal tendon bone tissue (Figure 7). Finally, the results of histomorphological staining showed that the gradient structure of the regenerated tendon bone tissue was close to that of the normal tissue, and the arrangement of collagen fibres was orderly (Figure 8).

There are also some limitations of this study. First, the animal model used in this study was an acute RCT model, and further exploration is needed for chronic RCTs and age‐related rotator cuff injuries. Second, due to the rapid degradation rate of tFNAs, this study used multiple injections into the joint cavity, which not only increased the risk of infection in the joint but also caused an uneven biological distribution. A controlled release system may be more beneficial for efficient use of the tFNAs. In addition, there is still a large gap between the mechanical properties of the repaired tissue in vivo and those of the normal tissue. Therefore, in our future work, we may need to study the combination of tetrahedra and scaffolds or biological meshes to better recover tendon‐bone function. In any case, tFNAs significantly promote regeneration of the tendon‐bone gradient microstructure, which provides a promising treatment strategy for RCTs.

## 5 CONCLUSION

In summary, our study demonstrated that tFNAs, novel nanomaterials, could reduce the assembly of macrophage inflammasomes in the inflammatory microenvironment, thereby reducing the release of inflammatory factors. We also found that tFNAs could protect BMSC osteogenic and chondrogenic differentiation under inflammatory conditions. We elucidated the regulatory effects of tFNAs on tendon‐associated protein expression levels in tenocytes following inflammatory stimulation. In addition, tFNAs were shown to improve tendon‐to‐bone healing in a rat acute RCT model. To the best of our knowledge, this study is the first report of the therapeutic effect of tFNAs in the context of rotator cuff injury, indicating that these nanomaterials might be promising tendon‐bone protective agents for RCT treatment.

## AUTHOR CONTRIBUTIONS

Pinxue Li, Liwei Fu as well as Chao Ning conducted the majority of the experiments, receiving assistance from Jiang Wu, Cangjian Gao performed the immunostaining and confocal microscopy experiments. Xiang Sui provided the essential substances and conducted the experiments involving animals. Pinxue Li assisted in overseeing and analysing data. The project was led by Yunfeng Lin, Shuyun Liu, Zhiguo Yuan, and Quanyi Guo and the paper was written by Pinxue Li with assistance from all of the authors.

## CONFLICT OF INTEREST STATEMENT

The authors assert that they possess no conflicting concerns.

## Supporting information


**Data S1:** Supporting Information

## Data Availability

The data that support the findings of this study are available from the corresponding author upon reasonable request.
