# Supplementary material for: Effect of tetrahedral framework nucleic acids on the reconstruction of tendon‐to‐bone injuries after rotator cuff tears
Source: Cell Prolif. 2024 Jan 28;57(6):e13605. doi: 10.1111/cpr.13605 (PMC11150141; doi:10.1111/cpr.13605)
Supplement: Supplementary file 1 — Data S1: Supporting Information [file CPR-57-e13605-s001.docx]

Supplementary Materials for

Effect of tetrahedral framework nucleic acids on the reconstruction of tendon-to-bone injuries after rotator cuff tears

Pinxue Li^1,2^^#^, Liwei Fu^1,2#^, Chao Ning^2#^, Jiang Wu^2#^, Zizheng Xu^1,2^, Zhiyao Liao^1,2^, Cangjian Gao^1^, Xiang Sui^2^, Yunfeng Lin^3^*, Shuyun Liu^2^*, Zhiguo Yuan ^4^*, Quanyi Guo^1,2^*

Correspondence to: yunfenglin@scu.edu.cn, [clear_ann@163.com](mailto:clear_ann@163.com), yzgad@163.com and doctorguo_301@163.com

1: School of Medicine, Nankai University, Tianjin 300071, People’s Republic of China

2: Institute of Orthopedics, Chinese PLA General Hospital; Beijing Key Laboratory of Regenerative Medicine in Orthopedics; Key Laboratory of Musculoskeletal Trauma & War Injuries PLA; No. 28 Fuxing Road, Haidian District, Beijing 100853, People’s Republic of China

3: State Key Laboratory of Oral Diseases, National Clinical Research Center for Oral Diseases, West China Hospital of Stomatology, Sichuan University, Chengdu 610041, People’s Republic of China

4: Department of Bone and Joint Surgery, Renji Hospital, School of Medicine, Shanghai Jiaotong University, Shanghai 200030, People's Republic of China.

**Corresponding Authors**

*E-mail: yunfenglin@scu.edu.cn (Y.-F.L.)

*E-mail: [clear_ann@163.com](mailto:clear_ann@163.com) (S.-Y.L.)

*E-mail: yzgad@163.com (Z.-G.Y.)

*E-mail: doctorguo_301@163.com (Q.-Y.G.)

**Author Contributions**

Pinxue Li, Liwei Fu, Chao Ning and Jiang Wu contributed equally to this paper.

**Supplementary Materials and Methods**

1.1 Cell culture

The study was approved by the Ethical Committee of PLA. The isolation and culture of BMSCs were performed according to previous research[1]. In short, the tibia and femur bones of SD rats were separated under sterile conditions. The bone marrow cavity was rinsed with phosphate-buffered saline (PBS) containing 1% penicillin streptomycin (Sigma, USA), drawn with a syringe, and rinsed repeatedly. The BMSCs isolate was centrifuged at 500 × g for 10 min at a concentration of 10% (v/v) FBS and 1% (v/v) penicillin‒streptomycin (GIBCO, Biosciences, Inc., Inc. Ireland) in basal medium (DMEM) and incubated at 37 °C containing 5% CO2, with the medium changed every two days. Once the density of BMSCs reached 80%, they were subcultured with trypsin (Sigma). BMSCs from the third to fourth passages were used in this experiment.

Tenocytes were isolated and cultured according to previous studies[2, 3]. In general, tendon tissue was dissected from r Achilles tendon of SD rats and washed with PBS supplemented with 1% penicillin streptomycin. The tendon tissue was then cut into small pieces (1 mm3) with ophthalmic scissors and digested with a sterile rotor for 1 h in Dulbecco's modified Eagle's medium (DMEM, Corning) containing 0.3% type I collagenase. After digestion, the medium was filtered with a 100 μm Cell Strainer, and the tenocytes were resuspended in DMEM containing 10% fetal bovine serum (FBS) and centrifuged. After centrifugation, the cells were resuspended again and transferred to 25 cm^2^ flasks (5% CO_2_, 37 °C). Once the density of BMSCs reached 80%, they were subcultured with trypsin (Sigma)

1.2 Trilineage-induced differentiation and flow cytometry experiment

We proved the adipogenic, osteogenic and chondrogenic differentiation potential of BMSCs through a trilineage-induced differentiation experiment. BMSCs at passage 2 were used in subsequent experiments. In the adipogenic experiment, 1X10^5^ BMSCs were cultured in a 6-well plate and MSC adipogenic differentiation medium (Cyagen Biosciences, Guangzhou, China) after 7 days of culture, BMSCs were fixed with paraformaldehyde, and the degree of adipogenesis was determined by Oil red O staining. Osteogenic induction culture was similar to adipogenic differentiation, but BMSCs were cultured in osteogenic differentiation medium (Cyagen Biosciences, Guangzhou) for 14 days and stained with alizarin red for chondrogenic differentiation. BMSCs (3X10^5^) were centrifuged at 250 g for 5 min in 15 mL Falcon tubes to form cell pellets. The pellets were maintained at 37 °C with 5% CO_2_ in basal media for 24 h, and each tube was nourished with chondrogenic differentiation medium (MSCgo^TM^, Biological Industries, Israel) and replenished every 3 days for 14 days. Finally, the pellets were prepared by the frozen section procedure and stained with Alcian blue.

A suspension of 1 × 10^6^ (1 mL) was placed in a centrifuge tube, BMSCs were identified using flow cytometry (Beckman Coulter, CytoFLEX). The antibodies for positive surface markers included CD 90-APC (BD Biosciences,561409) and CD105-PE/Cy7 (NB500-452PECY7), while the negative markers included CD 34-FITC (Novus, NB2-54355F) and CD 45RA-APC/Cy7 (BD Biosciences,561624).

1.3 Cellular uptake of tFNAs

To verify the uptake of tFNAs by RAW 264.7 cells, BMSCs and tenocytes, we modified tFNAs and ssDNA with cyanine3 (Cy3) and cocultured them with RAW 264.7 cells, BMSCs and tenocytes for 12 h. Subsequently, the RAW 264.7 cells, BMSCs and tenocytes were rinsed with PBS and fixed with 4% polyoxymethylene solution for 30 min. After that, RAW 264.7 cells, BMSCs and tenocytes were stained with 4′,6-diamidino-2-phenylindole (DAPI, 1:250, Life Technologies) for 5 min. Finally, images of all samples were captured with a fluorescence microscope (Nikon, Japan).

1.4 Effect of tFNAs on RAW 264.7 cells under inflammatory conditions

1.4.1 RT-qPCR

The expression of iNOS, CD206, IL-1β and IL-6 in RAW 264.7 cells under inflammatory conditions of different groups at 3 days was analyzed by a Cell Total RNA Isolation Kit (Foregene, Chengdu, China). We extracted total RNA from RAW 264.7 cells after 3 days of culture and converted total RNA to complementary DNA using 5 × RT Master Mix (Toyobo, Osaka, Japan). RT-PCR was performed on a StepOneTM Real-Time PCR system (Applied Biosystems, USA) using 2 × RealStar Green Fast mixture (Genstar, Beijing, China) according to the standard procedure. The primer sequences for iNOS, CD206, IL-1β and IL-6 and GAPDH are shown in Table S1. To detect the reliability of the primers, we established fusion curves for each reaction system, and there was no nonspecific amplification in the dissolution curves. Relative mRNA expression was normalized to that of the housekeeping gene GAPDH and calculated by using the 2^-ΔΔ^CT method.

1.4.2 Western blot

RAW 264.7 cells were transferred to 6-well plates and cultured of different groups for 3 days. Cell samples were digested with trypsin and centrifuged to detect proliferation-related proteins. Cellular protein extraction reagent (Beyotime, Shanghai, China) was used to extract RAW 264.7 cells proteins. The protein samples were incubated overnight with the following primary antibodies: anti-β-actin (1:5000, Immunoway, TX, USA), anti-TMS1/ACS (1:1500, Abcam, Cambridge, England), anti- NLRP3 (1:1500, Novus, NY, USA), anti-pro Caspase-1 + p10 + p12 (1:2000, Abcam, Cambridge, England) and anti- IL-1β (1:1500, Abcam, Cambridge, England). The samples were then incubated with the secondary antibody (Abcam, Cambridge, England) for 45 min. Subsequently, the protein bands were visualized using enhanced chemiluminescence.

1.5 Effect of tFNAs on osteogenic differentiation of BMSCs under inflammatory conditions.

1.5.1 Alizarin red staining

BMSCs were transferred to 6-well plates and cultured in osteogenic-induction medium (Cyagen Biosciences, Guangzhou, China) of different groups for 14 days. After 14 days of coculture in three different media, washed with PBS for three times, the BMSCs were fixed with paraformaldehyde for 30 minutes. Alizarin red staining was applied to dye the samples for a half hour at room temperature and a microscope was used to survey the calcium deposition.

1.5.2 RT-qPCR

The expression of genes related to the osteogenic differentiation (ALP, OPN and RUX2) of BMSCs of different groups at 1 days after culture was analyzed by a Cell Total RNA Isolation Kit (Foregene, Chengdu, China). We extracted total RNA from the BMSCs after 1 days of culture and converted total RNA to complementary DNA using 5 × RT Master Mix (Toyobo, Osaka, Japan). RT-PCR was performed on a StepOneTM Real-Time PCR system (Applied Biosystems, USA) using 2 × RealStar Green Fast mixture (Genstar, Beijing, China) according to the standard procedure. The primer sequences for ALP, OPN, RUX2 and GAPDH are shown in Table S1. To detect the reliability of the primers, we established fusion curves for each reaction system, and there was no nonspecific amplification in the dissolution curves. Relative mRNA expression was normalized to that of the housekeeping gene GAPDH and calculated by using the 2^-ΔΔ^CT method.

1.5.3 Western blot

BMSCs were transferred to 6-well plates and cultured of different groups for 3 days. Cell samples were digested with trypsin and centrifuged to detect proliferation-related proteins. Cellular protein extraction reagent (Beyotime, Shanghai, China) was used to extract BMSC proteins. The protein samples were incubated overnight with the following primary antibodies: anti-β-actin (1:5000, Immunoway, TX, USA), anti-OPN (1:1500, Abcam, Cambridge, England) and anti-RUNX2 (1:2000, Novus, NY, USA). The samples were then incubated with the secondary antibody (Abcam, Cambridge, England) for 45 min. Subsequently, the protein bands were visualized using enhanced chemiluminescence.

1.5.4 Immunofluorescence staining

We used immunofluorescence to examine the localization and expression of proteins related to osteogenic differentiation. The BMSCs were transferred to cell slides prepositioned in a 24-well plate and then cultured of different groups for 3 days. BMSCs were washed with PBS after delivery, and then the samples were fixed with paraformaldehyde for 30 min. Triton X-100 (0.5%) and immune blocking solution (Beyotime, Shanghai, China) were used to permeabilize and block BMSCs. After 3 washes with PBS, the samples were incubated with anti-OPN (1:200, Abcam, Cambridge, England) and anti-RUNX2 (1:200, Novus, NY, USA) overnight at 4 °C. Fluorescent secondary antibody was added and incubated for 2 h. FITC-phalloidin (Beyotime) was used to stain F-actin. Finally, the nuclei were stained with DAPI (1:1000, Life Technologies, CA, USA). Images were taken by a fluorescence microscope (Nikon, Japan).

1.6 Effect of tFNAs on chondrogenic differentiation of BMSCs under inflammatory conditions.

1.6.1 Pellet culture

The effect of tFNAs on the chondrogenic differentiation of BMSCs was investigated by cultured cartilage pellets under inflammatory conditions according to previous reports[4]. The BMSCs (4X10^5^) were centrifuged at 1300 rpm for 4 min in 15 mL tubes to form BMSC pellets. The pellets were cultured in basal medium for 24 h, after which each tube was supplied with chondrogenic differentiation medium (Cyagen, China) containing LPS (2 μg/mL), LPS (2 μg/mL) plus tFNAs (250 nM) and vehicle control, and the medium was replaced 2 to 3 times per week for 21 days. After the cartilage pellets were cultured in three different media, the degree of chondrogenic formation was estimated through H&E, Alcian blue, Safranin O, and type II collagen immunofluorescence staining.

1.6.2 RT-qPCR

The expression of genes related to the chondrogenic differentiation of pellets of different groups at 21 days was analyzed by a Cell Total RNA Isolation Kit (Foregene, Chengdu, China). We extracted total RNA from the pellets after 21 days of culture and converted total RNA to complementary DNA using 5 × RT Master Mix (Toyobo, Osaka, Japan). RT-PCR was performed on a StepOneTM Real-Time PCR system (Applied Biosystems, USA) using 2 × RealStar Green Fast mixture (Genstar, Beijing, China) according to the standard procedure. The primer sequences for Col II, Sox9, Aggrecan, Col I and GAPDH are shown in Table S1. To detect the reliability of the primers, we established fusion curves for each reaction system, and there was no nonspecific amplification in the dissolution curves. Relative mRNA expression was normalized to that of the housekeeping gene GAPDH and calculated by using the 2^-ΔΔ^CT method.

1.6.3 Western blot

After 21 days of culture, pellet samples were digested with trypsin and centrifuged to detect proliferation-related proteins. Cellular protein extraction reagent (Beyotime, Shanghai, China) was used to extract pellet proteins. The protein samples were incubated overnight with the following primary antibodies: anti-β-actin (1:5000, Immunoway, TX, USA), anti-Col II (1:1500, Abcam, Cambridge, England), anti-SOX-9 (1:1500, Abcam, Cambridge, England) and anti-Aggrecan (1:2000, Novus, NY, USA). The samples were then incubated with the secondary antibody (Abcam, Cambridge, England) for 45 min. Subsequently, the protein bands were visualized using enhanced chemiluminescence.

1.7 Effect of tFNAs on tenocytes protein expression under inflammatory conditions.

1.7.1 RT-qPCR

The genes expression of Col I and Col III of tenocytes of different groups at 1 days after culture was analyzed by a Cell Total RNA Isolation Kit (Foregene, Chengdu, China). We extracted total RNA from the BMSCs after 1 days of culture and converted total RNA to complementary DNA using 5 × RT Master Mix (Toyobo, Osaka, Japan). RT-PCR was performed on a StepOneTM Real-Time PCR system (Applied Biosystems, USA) using 2 × RealStar Green Fast mixture (Genstar, Beijing, China) according to the standard procedure. The primer sequences for Col I, Col III and GAPDH are shown in Table S1. To detect the reliability of the primers, we established fusion curves for each reaction system, and there was no nonspecific amplification in the dissolution curves. Relative mRNA expression was normalized to that of the housekeeping gene GAPDH and calculated by using the 2^-ΔΔ^CT method.

1.7.2 Western blot

Tenocytes were transferred to 6-well plates and cultured of different groups for 3 days. Cell samples were digested with trypsin and centrifuged to detect proliferation-related proteins. Cellular protein extraction reagent (Beyotime, Shanghai, China) was used to extract BMSC proteins. The protein samples were incubated overnight with the following primary antibodies: anti-β-actin (1:5000, Immunoway, TX, USA), anti-Col I (1:1500, Abcam, Cambridge, England), anti-TNMD (1:1500, Abcam, Cambridge, England) and anti-Col III (1:1500, Abcam, Cambridge, England). The samples were then incubated with the secondary antibody (Abcam, Cambridge, England) for 45 min. Subsequently, the protein bands were visualized using enhanced chemiluminescence.

1.7.3 Immunofluorescence staining

We used immunofluorescence to examine the localization and expression of Col I and Col III. The tenocytes were transferred to cell slides prepositioned in a 24-well plate and then cultured of different groups for 3 days. Tenocytes were washed with PBS after delivery, and then the samples were fixed with paraformaldehyde for 30 min. Triton X-100 (0.5%) and immune blocking solution (Beyotime, Shanghai, China) were used to permeabilize and block tenocytes. After 3 washes with PBS, the samples were incubated with anti-Col I (1:200, Abcam, Cambridge, England) and anti-Col III (1:200, Abcam, Cambridge, England) overnight at 4 °C. Fluorescent secondary antibody was added and incubated for 2 h. FITC-phalloidin (Beyotime) was used to stain F-actin. Finally, the nuclei were stained with DAPI (1:1000, Life Technologies, CA, USA). Images were taken by a fluorescence microscope (Nikon, Japan).

1.8 Animal models

Animal experiments were approved by the Institutional Animal Care and Use Committee at PLA General Hospital. Forty male Sprague-Dawley rats (220-240g) were randomly allocated into three groups: the sham group (n=8), the negative control group (n=16) and the tFNAs group (n=16). The surgical procedures are described briefly as follows, rats were anesthetized and the deltoid muscles were exposed after skin disinfection. Then the deltoid of right shoulder was cut and the supraspinatus was exposed, the supraspinatus tendon was marked with absorbable suture, the tendon-to-bone junction was transected, the greater tubercle of humerus was grinded with a blade to stimulate the bone marrow, and an Angle puncture needle was used to drill a tunnel at the greater tuberosity of the humerus. Finally, the supraspinatus tendon and the greater tuberosity were re-sutured with absorbable suture. After operation, the tFNAs group received intra-articular injection of 50 μL tFNAs (250 nM) every other day for 7 times for a total of 2 weeks, while the control group was injected with the same volume of normal saline. The samples were harvested after euthanasia at 6 and 12 weeks after surgery.

1.9 Small-animal MRI scanning

After sacrifice, the right operation shoulders from 3 specimens in each group at two different time points were examined via animal MRI system (Agilent Technologies). Coronal T2- weighted sequences with fat suppression images were acquired along the scapular plane and applied to evaluate the tendon-to-bone interface repair effects involving thickness, continuity, and signal intensity.

1.10 Microcomputed tomography (micro-CT) analysis

After the macro evaluation, the samples of the three groups were scanned in the General Electric (GE) Explorer Locus SP system (GE, Boston, MA, USA). After scanning, the bone mineral density (BMD) and bone volume/tissue volume (BV/TV) of the ROI of each sample were used to evaluate subchondral bone regeneration.

1.11 Biomechanical testing

We used a biomechanical testing machine (Bose, 5100) to detect the mechanical properties of the samples at 6 and 12 weeks after surgery. The sample was fixed in a gripping device and mounted on a mechanical testing machine, and the sample was gradually stretched at a speed of 3mm/min until fracture. Finally, the ultimate load and stiffness were calculated according to the force-displacement curve

1.12 Histological evaluation and semiquantitative histological scoring.

Rotator cuff samples from all groups were collected and fixed in paraformaldehyde for 5 days, decalcified for 2 months and trimmed continuously during the decalcification process. After decalcification, the samples were dehydrated, embedded in paraffin and sliced to a thickness of 5 μm. Sections were stained with H&E, safranin O, and Sirius red according to standard procedures. The procedure for the immunohistochemical staining of type II collagen was as follows. After the section was dewaxed and washed, endogenous peroxidase was removed with hydrogen peroxide. Then, TritonX-100 was used for permeabilization. The samples were washed with PBS, blocked, and then incubated with anti-collagen II primary antibody (1:200, DSHB, IA, USA) overnight at 4 °C. Finally, an immunohistochemical secondary antibody was added, and a chromogenic agent was used. The slides were observed and photographed under a microscope. All images were given to a researcher with extensive experience in the histopathology who did not know the groups to score according to the histologic grading of the tendon-bone insertion site and tendon-maturing score (Table S2-3).

1.13 Statistical analysis

Differences among groups were compared with One-way ANOVA or Student’s t-test using SPSS 18.0 statistical software. All data are shown as the mean ± standard deviation (SD), and *p < 0.05 represents the threshold for statistical significance.

**Results**

BMSCs were isolated, cultured and demonstrated multidirectional differentiation ability (Figure S1). The cultured BMSCs showed a uniform, slender, spindle shape under the microscope (Figure S1A). After 14 days of culture in osteogenic induction medium, Alizarin red staining showed a large amount of matrix calcification with calcium nodule formation (Figure S1B), proving that the BMSCs were capable of osteogenic differentiation. After 7 days of culture in adipogenic induction medium, a large number of rounds, red-stained lipid droplets were observed by Oil red-O staining (Figure S1C). After 14 days of culture in chondrogenic induction medium, Alcian blue staining was positive (Figure S1D), indicating that the 3D cell pellets were rich in proteoglycans. The above experimental results confirm that BMSCs are capable of multilineage differentiation.

The flow cytometry results (Figure 2) of BMSCs showed that the cells expressed the MSC surface markers CD90 (99.98%) and CD105 (99.52%) and did not express the hematopoietic cell markers CD34 (0.17%) and CD45RA (0.92%). These data indicated that the isolated BMSCs had good homogeneity, without hematopoietic and endothelial cells, and had good MSCs characteristics.

**Supplementary Figures and Tables**


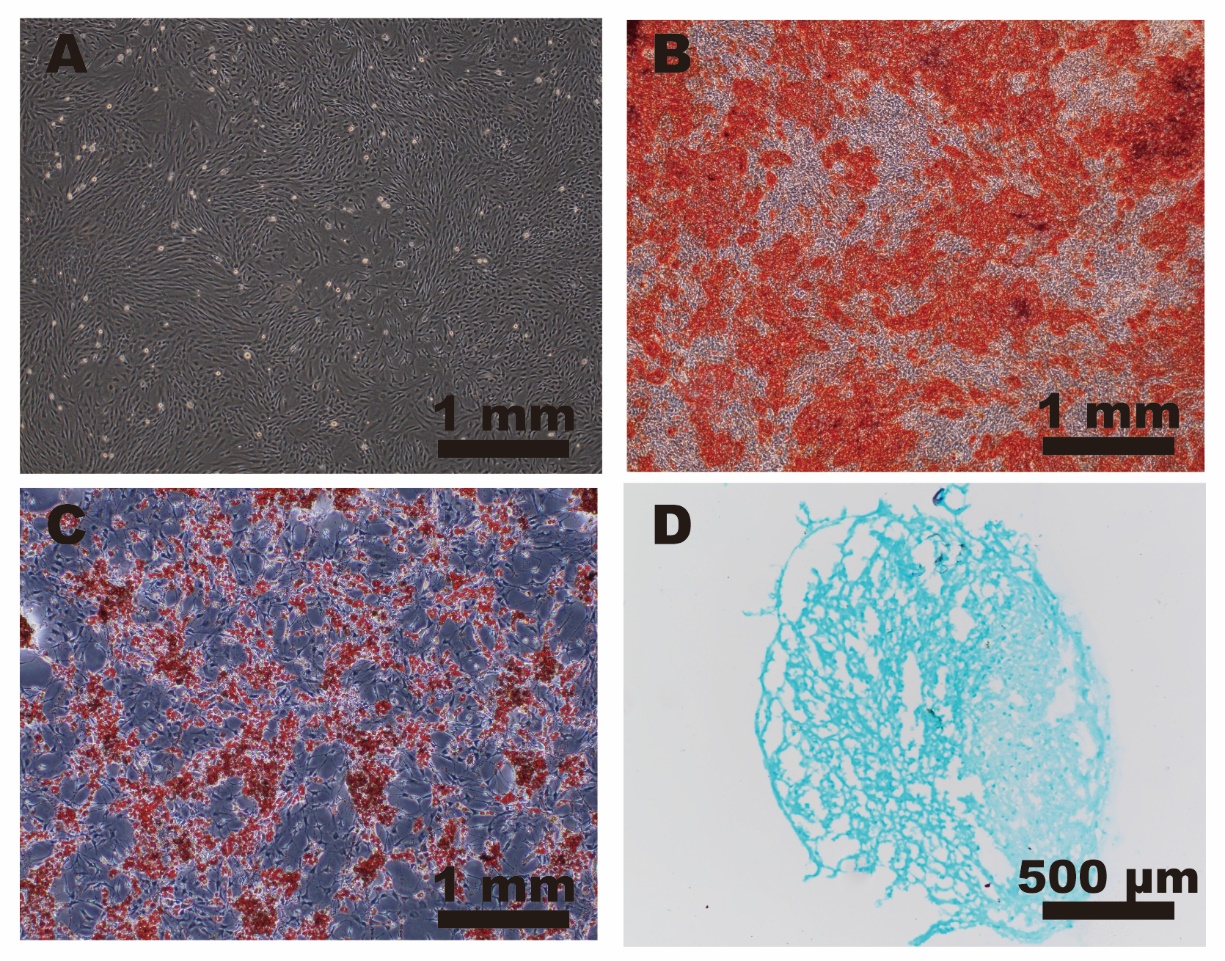


**Figure S1. (A) Morphological observation of BMSCs. (B) Osteogenic differentiation of BMSCs stained with Alizarin red. (C) BMSCs differentiated into fat and stained with Oil red O. (D) BMSCs differentiated into cartilage and stained with Alcian blue.**

**
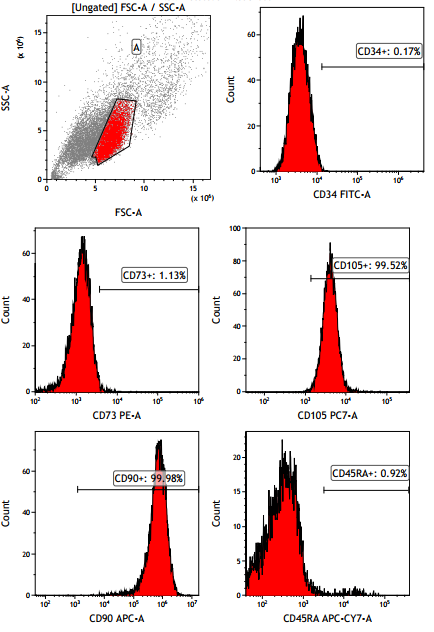
**

**Figure S2.** **Flow cytometry of surface makers of BMSCs.**


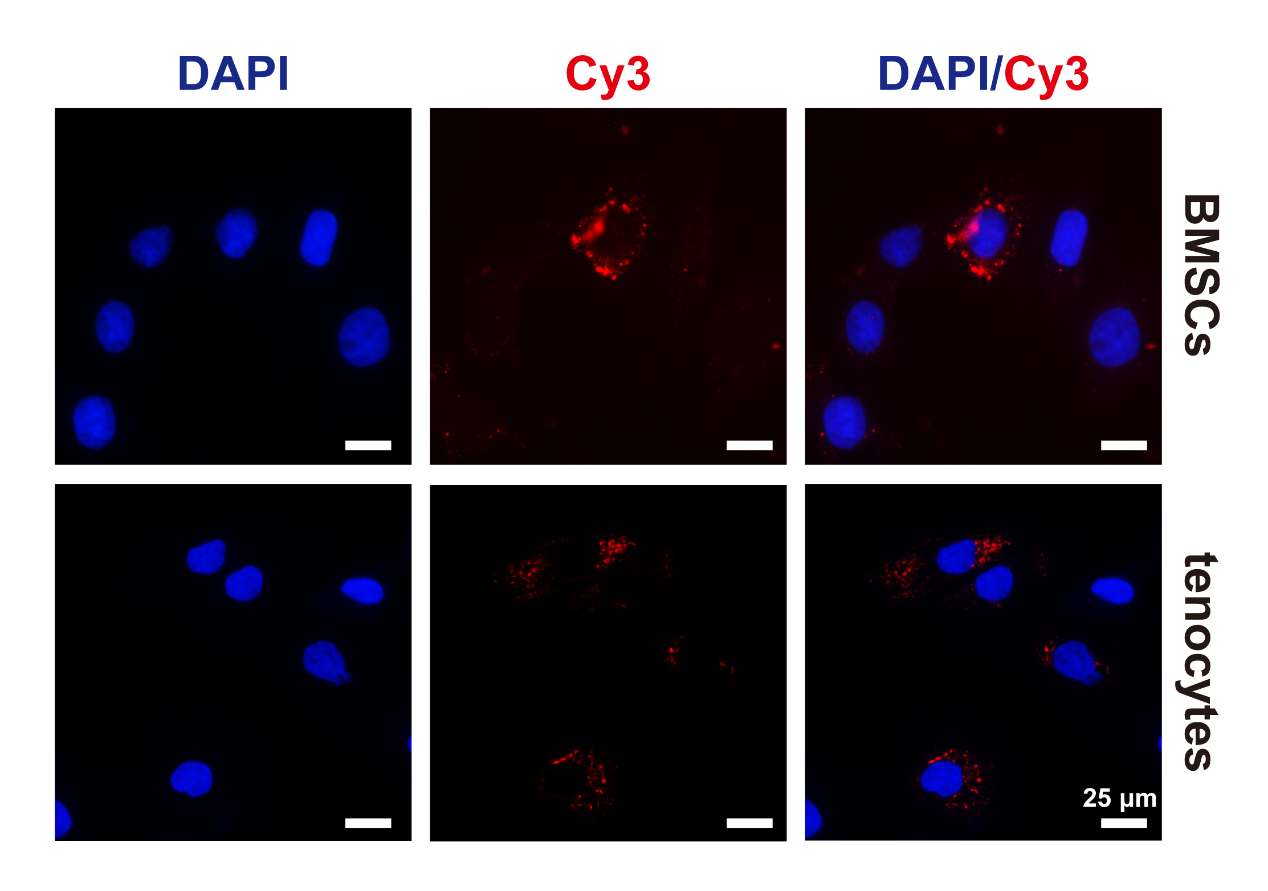


**Figure S3.** **Cellular uptake of tFNAs of BMSCs and tenocytes.**

**Table S1.** Primer sequences for quantitative RT-PCR.

| **Gene** | **Primer** | **Primer Sequence (5' to 3')** |
| --- | --- | --- |
| *iNOS (Mouse)* | Forward | GTTCTCAGCCCAACAATACAAGA |
| *iNOS (Mouse)* | Reverse | GTGGACGGGTCGATGTCAC |
| *CD206* *(Mouse)* | Forward | ATGGATGTTGATGGCTACTGG |
| *CD206* *(Mouse)* | Reverse | TTCTGACTCTGGACACTTGC |
| *IL-1β (Mouse)* | Forward | TACAGGCTCCGAGATGAACA |
| *IL-1β (Mouse)* | Reverse | AGGCCACAGGTATTTTGTCG |
| *IL-6 (Mouse)* | Forward | CTGCAAGAGACTTCCATCCAG |
| *IL-6 (Mouse)* | Reverse | AGTGGTATAGACAGGTCTGTTGG |
| *GAPDH* *(Mouse)* | Forward | CTTTGTCAAGCTCATTTCCTGG |
| *GAPDH* *(Mouse)* | Reverse | TCTTGCTCAGTGTCCTTGC |
| *ALP (Rat)* | Forward | ATCTTTGGTCTGGCTCCCATG |
| *ALP (Rat)* | Reverse | TTTCCCGTTCACCGTCCAC |
| *OPN (Rat)* | Forward | CACTCCAATCGTCCCTACA |
| *OPN (Rat)* | Reverse | CTTAGACTCACCGCTCTTCAT |
| *RUNX 2 (Rat)* | Forward | AGGGACTATGGCGTCAAACA |
| *RUNX 2 (Rat)* | Reverse | GGCTCACGTCGCTCATCTT |
| *Col II (Rat)* | Forward | GAGTGGAAGAGCGGAGACTACTG |
| *Col II (Rat)* | Reverse | GTCTCCATGTTGCAGAAGACTTTCA |
| *SOX-9 (Rat)* | Forward | CCAGCAAGAACAAGCCACAC |
| *SOX-9 (Rat)* | Reverse | CTTGCCCAGAGTCTTGCTGA |
| *Aggrecan (Rat)* | Forward | CTAGCTGCTTAGCAGGGATAACG |
| *Aggrecan (Rat)* | Reverse | GATGACCCGCAGAGTCACAAAG |
| *Col I (Rat)* | Forward | TGGTGAGACGTGGAAACCTG |
| *Col I (Rat)* | Reverse | CTTGGGTCCCTCGACTCCTA |
| *Col III (Rat)* | Forward | ACACCTGCTCCTGTCATTCC |
| *Col III (Rat)* | Reverse | AAGACCAGGGTCGCCATTTCAAGACCAGGGTCGCCATTTC |
| *GAPDH (Rat)* | Forward | GAAGGTCGGTGTGAACGGATTTG |
| *GAPDH (Rat)* | Reverse | CATGTAGACCATGTAGTTGAGGTCA |

**Table S2.** Histologic grading of the tendon-bone insertion site.

| Histologic grading of the tendon-bone insertion site | | | | |
| --- | --- | --- | --- | --- |
|  | 0 | 1 | 2 | 3 |
| Collagen fiber continuity | 0%–25 % of proportion | 25%–50 % of proportion | 50%–75 % of proportion | 75%–100 % of proportion |
| Collagen fiber oriented parallel | 0%–25 % of proportion | 25%–50 % of proportion | 50%–75 % of proportion | 75%–100 % of proportion |
| Collagen fiber density | very loose | loose | dense | very dense |
| Vascularity | absent or minimally present | mildly present | moderately present | severe or markedly present |
| Cellularity | absent or minimally present | mildly present | moderately present | severe or markedly present |

**Table S3.** The tendon-maturing score.

| Modified tendon-maturing score | | | | |
| --- | --- | --- | --- | --- |
|  | 1 | 2 | 3 | 4 |
| Cellularity | Marked | Moderate | Mild | Minimal |
| Proportion of cells resembling tenocytes | <25% | 25-50% | 50-75% | >75% |
| Proportion of cells oriented in parallel | <25% | 25-50% | 50-75% | >75% |
| Proportion of fibers of large diameter characteristic of mature tendon fibers | <25% | 25-50% | 50-75% | >75% |
| Proportion of fibers orients in parallel | <25% | 25-50% | 50-75% | >75% |

**REFERENCES**

[1] L. Wu, Y. Jia, Y. Sui, S. Ao, Y. Wang, Y. Liu, W. Xu, H. Zhang, H. Zhang, J. Mao, X. Yang, H. Leng, The effect of BMP2/Smads pathway mediating platelet-rich fibrin on rat bone mesenchymal stem cells, European review for medical and pharmacological sciences 26(15) (2022) 5413-5421.

[2] X. Zhou, J. Li, A. Giannopoulos, P. Kingham, L. Backman, Secretome from In Vitro Mechanically Loaded Myoblasts Induces Tenocyte Migration, Transition to a Fibroblastic Phenotype and Suppression of Collagen Production, International journal of molecular sciences 22(23) (2021).

[3] Y. Ren, S. Zhang, Y. Wang, D. Jacobson, R. Reisdorf, T. Kuroiwa, A. Behfar, S. Moran, S. Steinmann, C. Zhao, Effects of purified exosome product on rotator cuff tendon-bone healing in vitro and in vivo, Biomaterials 276 (2021) 121019.

[4] W. Song, Z. Ma, C. Wang, H. Li, Y. He, Pro-chondrogenic and immunomodulatory melatonin-loaded electrospun membranes for tendon-to-bone healing, J Mater Chem B 7(42) (2019) 6564-6575.
